# Five‐year‐old children value reasons in apologies for belief‐based accidents

**DOI:** 10.1111/cdev.13893

**Published:** 2023-01-24

**Authors:** Owen Waddington, Marina Proft, Keith Jensen, Bahar Köymen

**Affiliations:** ^1^ Division of Psychology, Communication and Human Neuroscience, School of Health Sciences University of Manchester Manchester UK; ^2^ Georg‐Elias‐Müller‐Institute for Psychology Georg‐August‐University Göttingen Göttingen Germany

## Abstract

Accidents can be intent‐based (unintended action‐unintended outcome) or belief‐based (intended action‐unintended outcome). As compared to intent‐based accidents, giving reasons is more crucial for belief‐based accidents because the transgressor appears to have intentionally transgressed. In Study 1, UK‐based preschoolers who were native English speakers (*N* = 96, 53 girls, collected 2020–2021) witnessed two intent‐based or belief‐based accidents; one transgressor apologized, the other apologized with a reason. Five‐year‐olds, but not 4‐year‐olds, favored the reason‐giving transgressor following a belief‐based accident but not an intent‐based accident (where an apology sufficed). In Study 2, 5‐year‐olds (*N* = 48, 25 girls, collected 2021) distinguished between “good” and “bad” reasons for the harm caused. Thus, 5‐year‐old children recognize when reasons should accompany apologies and account for the quality of these reasons.

**Abbreviations**
ANCOVA: analysis of covariance
ANOVA: analysis of variance
GLM: generalized linear model

Following an offense, a common approach to restore relationships is for the offender to apologize. Apologies show the offender regrets their actions (Leary, 1996), cares about the victim's feelings (Schleien et al., 2010) and wishes to make amends (Schlenker, 1980). Preschool children already grasp the mitigating function of apologies. They view apologetic transgressors as being more remorseful (Smith & Harris, 2012), likable (Banerjee et al., 2010) and “just” (Irwin & Moore, 1971), and forgive apologetic transgressors more than unapologetic transgressors (Oostenbroek & Vaish, 2019).

Apologies are perceived differently according to the kind of transgression they proceed. Research has mostly focused on apologies following violations in which the intention and the outcome are matched. These include *intentional transgressions* wherein the act and outcome are intended (e.g., intentionally smashing someone's cup), and *accidental transgressions* wherein the act and outcome are unintended (e.g., tripping and breaking someone's cup). Apologies, even more elaborate ones, are less effective following intentional harm, with blame and punishment still being issued (Darby & Schlenker, 1982, 1989; Ohbuchi & Sato, 1994) and the apology itself more likely to be rejected (Struthers et al., 2008). Apologies following accidental harm, on the other hand, typically engender forgiveness in adults and children alike (Oostenbroek & Vaish, 2019; see Yucel & Vaish, 2021 for a review).

A less explored kind of transgression, however, are *belief‐based accidents* in which the intention and outcome do not match. An individual might deliberately eat someone's sandwich by falsely believing that it was theirs. The act of eating the sandwich is intended, but the outcome (i.e., stealing somebody's food) is not. Unlike accidental transgressions, where the unintentional nature of the transgression is observable (e.g., the transgressor's surprised face, gasps) and in the common ground of the victim and transgressor (Köymen et al., 2016; Mammen et al., 2018; see also Bohn & Köymen, 2018), belief‐based accidents result in different interpretations of the offense. From the victim's perspective, the transgression appears intentional based on the intended action, whereas the transgressor considers it an accident due to the unintended outcome. Thus, giving reasons (e.g., “I thought the sandwich was mine”) for belief‐based accidents is crucial to clarify that the harm was unintended, while reasons for accidental transgressions are redundant.

Children tend to evaluate transgressors more positively if they have a “good” reason for their misbehavior. Schmidt et al. (2016) found that 8‐year‐old children excused a greedy puppet if that puppet needed or deserved the resources, but not if she simply wanted more. Five‐year‐olds judged that it was more acceptable to break a promise for prosocial reasons (e.g., to help someone) than for selfish reasons (e.g., to play a more exciting game; Kanngiesser et al., 2021; Mammen et al., 2019).

Preschoolers also feature the mental states of transgressors in their normative judgments. Although 3‐ to 5‐year‐olds were thought to focus mostly on the extent of a harm (i.e., the outcome) when evaluating others' moral acts (Piaget, 1932; Zelazo et al., 1996), recent evidence shows that they also account for the actor's intentions (Margoni & Surian, 2020; Nobes et al., 2009; Vaish et al., 2010). With age, children increasingly judge a transgressor less according to outcome and more according to whether she was well‐ or ill‐intentioned (Cushman et al., 2013; Li et al., 2017; Nobes et al., 2016, 2017). This “outcome‐to‐intent” shift in children's moral reasoning, however, may not apply universally. In so‐called “opacity of mind” cultures (e.g., Fiji), individuals are known to be less mind‐minded in that they mostly disregard intentions and, instead, focus on outcomes when judging transgressions (Barrett et al., 2016; McNamara et al., 2019).

Studies tapping into children's understanding of belief‐based accidents, however, show a later developing competence around age 7 due to the conflicting action and outcome information (Helwig et al., 2001; Killen et al., 2011). After witnessing a series of belief‐related mistakes, Proft and Rakoczy (2019) found 5‐ and 7‐year‐old children's judgments focused more on the intended action than on the unintended outcome. Only when primed for the intentional structure of such acts (i.e., whether the agent had intentionally caused the outcome) did 5‐year‐olds evaluate belief‐based accidents similarly to intent‐based ones. A similar pattern is found when children themselves are the victims of belief‐based accidents. Participants aged 5‐ to 10‐years‐old were told that another child had unknowingly scribbled over several desirable coloring sheets that were meant for them, and was sorry for having done so. Younger children forgave this remorseful transgressor less often than older children, suggesting that their judgments emphasized the intended action over the unintended outcome (Amir et al., 2021).

While children recognize the mitigating function of apologies and reasons for intentional and accidental transgressions, whether children attend to the *presence* and the *quality* of reasons that accompany apologies for belief‐based accidents is not known. Across two preregistered studies, we investigated whether 4‐ and 5‐year‐olds consider reason‐giving to be necessary when apologizing for belief‐based accidents (Study 1) and whether 5‐year‐olds evaluate the quality of these reasons (Study 2). We focused on these age groups due to children's sensitivity to others' mental states being present from age 4 (Wellman et al., 2001) but improves at age 5 to include normative judgments (Proft & Rakoczy, 2019).

## STUDY 1

In Study 1, 4‐ and 5‐year‐olds observed two transgressors rip a third‐party's picture. Both apologized, but one also offered a reason for her transgression. In the belief‐based accident condition, both transgressors ripped the pictures intentionally (with happy expressions). The reason‐giving transgressor said, “I'm sorry, I thought this was my picture”; the other only apologized. In the intent‐based accident condition, both transgressors ripped the pictures accidentally (with shocked expressions). The reason‐giving transgressor said, “I'm sorry, I was trying to see the picture better”; the other only apologized. Children then selected the transgressor they would rather help, play with, and trust with their toy.

As part of our confirmatory hypotheses, we expected children, especially 5‐year‐olds, to prefer the reason‐giving transgressor in the belief‐based accident condition since the reason clarified that the outcome was unintended (Proft & Rakoczy, 2019). In the intent‐based accident condition, we expected both ages to show no preference for either transgressor since it was already in common ground (through facial expressions) that the transgressions were unintended, and thus providing a reason was not necessary (Köymen et al., 2016). We also anticipated the trust partner‐choice question (i.e., who should mind the child's toy) to be the most diagnostic because of its resemblance to the stories depicting the transgressors. As part of our exploratory hypotheses, we investigated whether there was an age or condition difference in the way children justified their preferences.

### Method

The procedure, hypotheses, sample size, exclusion criteria, and statistical analyses were preregistered (https://osf.io/jahzx).

#### Participants

Forty‐eight 4‐year‐olds (*M* = 4;6 [years; months], range = 4;0–4;11, 28 girls) and forty‐eight 5‐year‐olds (*M* = 5;4, range = 5;0–6;0, 25 girls) participated in the study and were randomly assigned to one of two conditions. Data collection took place between July 2020 and February 2021. The mean age of 4‐year‐olds differed by condition (*t*(46) = 2.26, *p* = .029, *d* = 0.65), with participants in the belief‐based accident condition being older (*M* = 4;7, SD = 0;3) than the participants in the intent‐based accident condition (*M* = 4;5, SD = 0;3). The mean age of 5‐year‐olds did not differ between the belief‐based accident (*M* = 5;5, SD = 0;4) and intent‐based accident (*M* = 5;4, SD = 0;3) conditions (*t*(46) = 0.66, *p* = .516, *d* = 0.19). Two additional 4‐year‐olds were excluded due to inattentiveness and failing to answer over half of the warm‐up trials correctly. Children who were native speakers of English were recruited from a database covering northwest England. We did not collect individual data about participants' socioeconomic or ethnic background, but families in this database come from predominantly White, middle‐class backgrounds. Informed parental consent and verbal child assent were obtained before participation in the study.

#### Materials

In the first set of warm‐up trials (5 trials), children were shown two objects on the screen and asked to identify one. In the second set, (4 trials) children heard four vignettes depicting two characters and were asked which was more likeable. In the test trial, children witnessed a story of two transgressors who apologized for their transgressions, with 15 PowerPoint slides containing still images and pre‐recorded narration (Appendix A). The pre‐recorded narration was voiced by the male experimenter, and the transgressors' dialogue by two female speakers.

#### Procedure

Children and their parents joined an online Zoom meeting in which the experimenter (E) screen‐shared a PowerPoint presentation. Parents were asked to confirm that the presentation was showing correctly (e.g., that it was not windowed). They could then choose whether or not to remain present during testing. If parents chose to stay, they were asked to sit behind their child and to not engage with them nor comment on the situation. In the first set of warm‐up trials, children saw slides containing two objects (e.g., flower and ball) and were asked to locate one by pointing to ensure the slides were correctly displayed. In the second warm‐up, children were presented with four vignettes depicting a “good” and “bad” character to check whether they preferred “nicer” individuals. In vignette 1, a boy hurt himself. One friend helped him; the other did not. Children were asked: “which friend do you like more?”. The next three vignettes followed a similar structure (Appendix A). Across all vignettes, the character presented first was counterbalanced.

In a single test trial, each child saw two transgressions in counterbalanced order. Each began with the victim, Tom, and one transgressor, Lisa, drawing matching pictures which were then placed on a table. The transgressor picked up a picture and ripped it. In the belief‐based accident condition, the transgressor laughed and smirked during her transgression, highlighting that the act was intended. Lisa then apologized: “I'm sorry.” Next, the victim drew matching pictures with the other transgressor, Poppy. The second transgression was identical to the first, except that Poppy apologized and gave a reason for her transgression: “I'm sorry, I thought this was my picture.” The identity of the reason‐giving transgressor was also counterbalanced.

Children then answered three partner‐choice questions in fixed order. For the help question, the transgressors were missing the same piece in their puzzles. E said: “They both ripped up Tom's pictures, but who would you like to help? Lisa or Poppy?” For the play question, both transgressors held a ball and children decided who they would rather play with. For the trust question, a toy (said to be the participant's) was positioned between the transgressors and children decided who should look after it. Finally, children were asked why they favored their preferred transgressor (i.e., the transgressor chosen in 2 or more questions): “You seemed to like X more, why?”, and their disfavored transgressor: “You seemed to like Y less, why?” If children referred only to the apology, an additional prompt was used to elicit more detailed reasons: “Did she say anything else?”

The intent‐based accident condition was identical, except that the transgressors gasped with shocked expressions during their transgressions, highlighting that each act was unintended. One transgressor apologized; the other apologized with a reason: “I'm sorry, I was trying to see the picture better.”

Sessions were recorded for coding purposes and lasted approximately 15–20 min.

#### Coding

We coded for which transgressor children favored in each partner‐choice question. We then identified children's ‘reason‐related justifications’ for their preferences, which referred to the transgressor's reason (e.g., “She thought it was hers”) or lack thereof (e.g., “She said sorry, but she didn't say why”). A second coder, blind to predictions, coded 25% of the data (24 children). Agreement was *κ* = .90.

### Results

As part of the preliminary analyses, we analyzed whether children's performance in the warm‐up trials (in which children identified the “nicer” character) varied by age group and condition. For 4‐year‐olds, the mean number of trials in which they correctly chose the “nicer” character was 3.79 in the belief‐based accident condition and 3.58 in the intent‐based accident condition (out of four trials). For 5‐year‐olds, the mean number of trials answered correctly was 3.83 in the belief‐based accident condition and 3.96 in the intent‐based accident condition. Children's warm‐up performance did not differ between conditions (4‐year‐olds: *t*(46) = 1.23, *p* = .224, *d* = 0.36; 5‐year‐olds: *t*(46) = 1.17, *p* = .248, *d* = 0.34).

**FIGURE 1:**
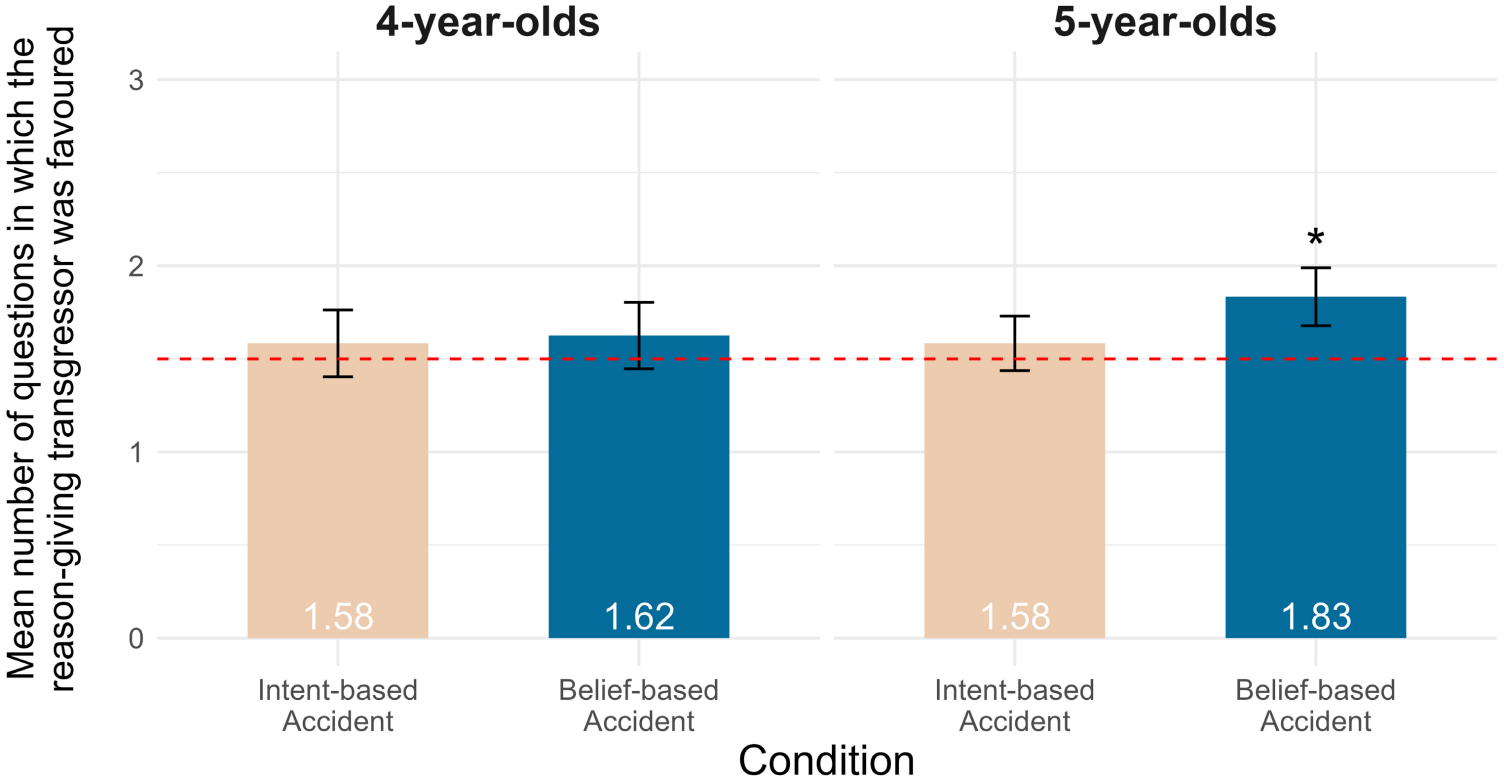
Mean number of partner‐choice questions in which the reason‐giving transgressor was favored by age group and condition. The red line represents chance, and error bars show standard error. *p < .05 compared to chance.

We then ran four analyses: three on children's preferences (preregistered) and one on children's justifications (exploratory). First, to identify children's overall preferences between the two transgressors, we conducted four one‐sample *t*‐tests (for each age group and condition) and compared the number of times children preferred the reason‐giving transgressor (0–3) to chance (1.5). Four‐year‐olds showed no preference for either transgressor in the intent‐based (*t*(23) = 0.46, *p* = .647, *d* = 0.09) nor belief‐based accident conditions (*t*(23) = 0.70, *p* = .491, *d* = 0.14). Five‐year‐olds showed a significant preference for the reason‐giving transgressor in the belief‐based accident condition (*t*(23) = 2.14, *p* = .043, *d* = 0.44), but not in the intent‐based accident condition (*t*(23) = 0.57, *p* = .575, *d* = 0.12; Figure 1).

Second, we compared children's preferences across conditions using a between‐subjects analysis of covariance (ANCOVA). The response variable was the number of times children preferred the reason‐giving transgressor. Predictors included: age (treated as a continuous covariate) and condition (intent‐based, belief‐based), their interaction, and gender. There was no significant interaction (*F*(1, 91) = 0.30, *p* = .588, $\eta_{p}^{2}$ = .003) nor main effects (*F*s(1, 91) < 0.72, *p*s > .397, $\eta_{p}^{2}s$ < .008; see Supplementary Materials A for the output summary), suggesting there were no age or condition differences in how often children favored the reason‐giving transgressor.

**FIGURE 2:**
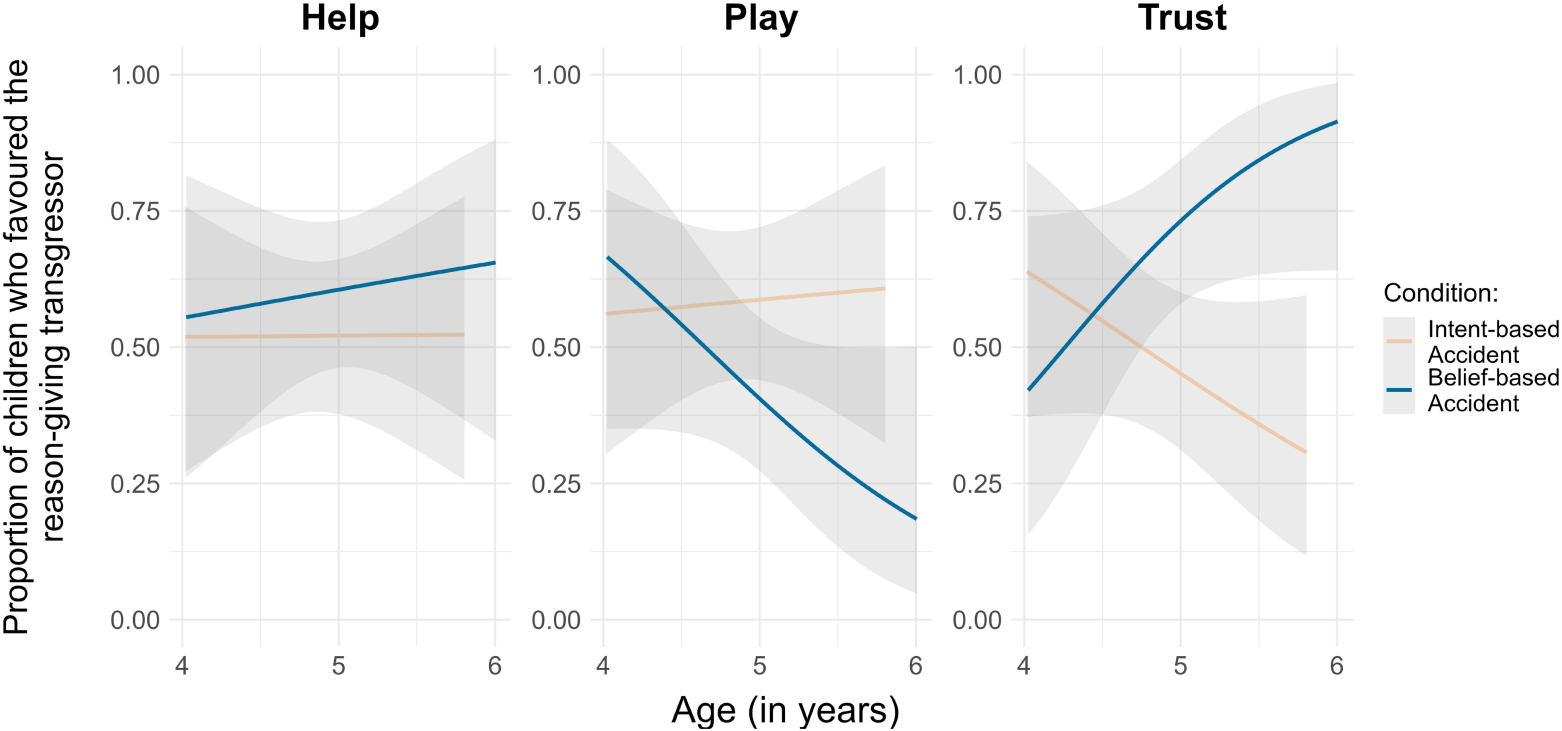
Children's preferences for the reason‐giving transgressor across the help, play and trust questions by age and condition. Lines represent fitted data with 95% confidence intervals.

Third, to determine children's preferences in each partner‐choice question, we ran three generalized linear models (GLMs) with binomial error distribution. The response variable was the binary measure of whether the child chose the reason‐giving transgressor. The full model included the predictors: age (treated as a continuous variable), condition, their interaction, and gender. The null model included gender only. The full model did not improve the fit for the help question (*χ* ^2^ = 0.69, df = 3, *p* = .875) nor the play question (*χ* ^2^ = 6.11, df = 3, *p* = .107). For the trust question, the full model improved the fit (*χ* ^2^ = 10.16, df = 3, *p* = .017). The interaction between age and condition was significant (*χ* ^2^ = 5.40, df = 1, *p* = .020; see Supplementary Materials B for the model summary). Five‐year‐olds were more likely to trust the reason‐giving transgressor in the belief‐based accident condition than in the intent‐based accident condition. Four‐year‐olds preferred each transgressor equally often across both conditions (Figure 2).

**FIGURE 3:**
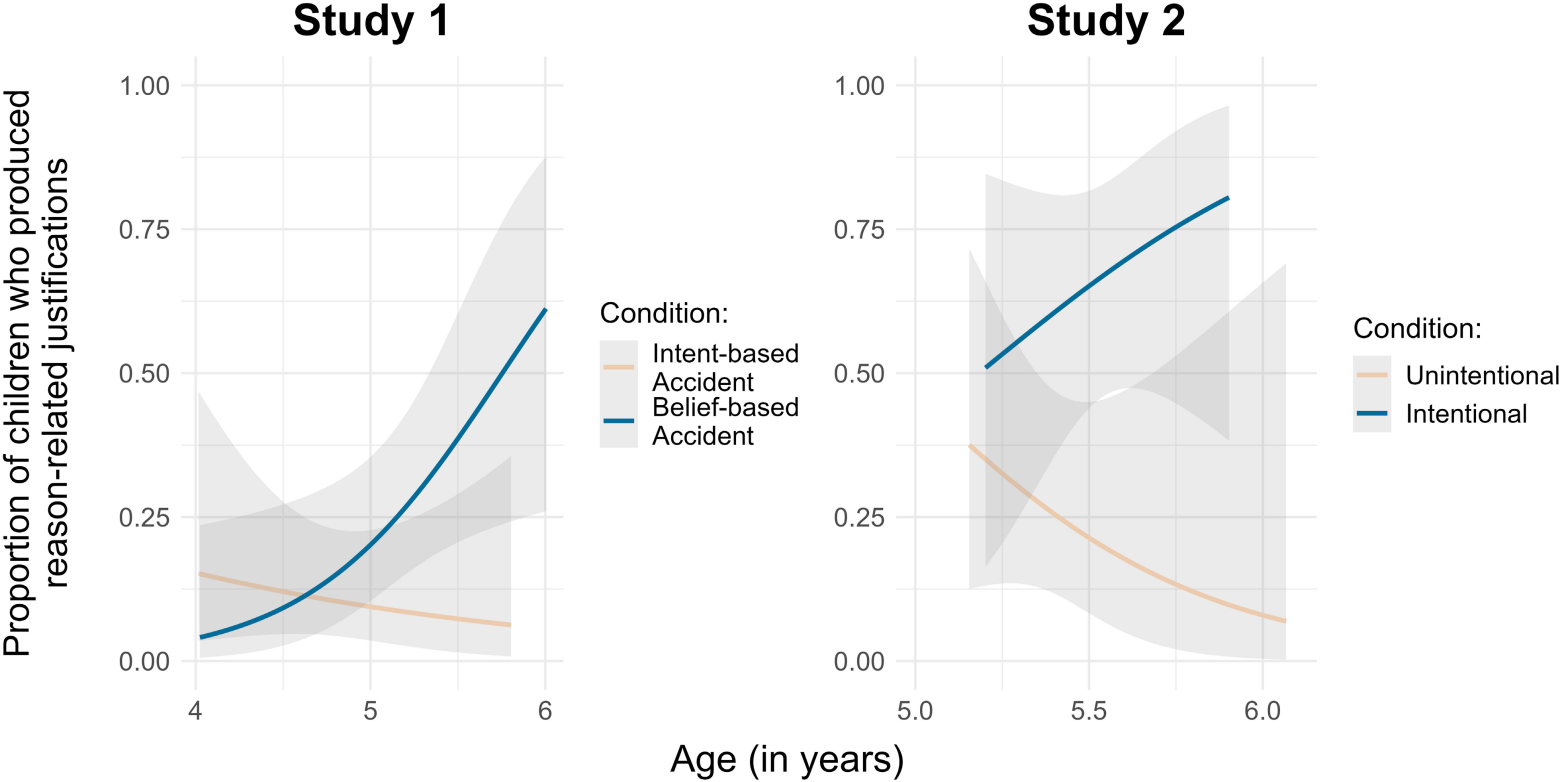
Children's justifications for their preferences across Study 1 and Study 2 by age and condition. Lines represent fitted data with 95% confidence intervals.

Finally, to investigate children's justifications for their preferences, we fitted a GLM with binomial error distribution. The response variable was the binary measure of whether children produced a reason‐related justification. The models were the same as the previous GLMs. The full model improved the fit (*χ* ^2^ = 9.87, df = 3, *p* = .020). The interaction between age and condition was significant (*χ* ^2^ = 4.56, df = 1, *p* = .033; see Supplementary Materials C for the model summary). Five‐year‐olds were more likely to give reason‐related justifications in the belief‐based accident condition than in the intent‐based accident condition, whereas 4‐year‐olds provided reason‐related justifications equally infrequently across both conditions (Figure 3).

In our preregistered analyses, we did not include order as a predictor variable. Subsequent analyses revealed, however, that the order in which the target transgressor was presented to children had no effect on the results obtained (see Supplementary Materials D for the full output summary). As such, we opted to report our analyses as outlined in our preregistrations. All statistical analyses were run in R (R Core Team, 2022).

### Discussion

For belief‐based accidents, 5‐year‐olds, but not 4‐year‐olds, preferred the reason‐giving transgressor over the transgressor who only apologized. This preference was largely driven by the trust question (i.e., which transgressor should look after the child's toy). Moreover, 5‐year‐olds referred to the reason provided in the belief‐based accident condition, excusing the reason‐giving transgressor (“She thought it was hers”) or condemning the apology‐only transgressor (“She said sorry, but she didn't say why”). Thus, at age 5, children only excused the belief‐based transgressor when she provided a reason which clarified that the harm was not intended.

Children did not simply favor the transgressor who had the most to say (Mercier et al., 2014). If this were the case, 5‐year‐olds would have shown a preference for the reason‐giving transgressor across both conditions. Rather, a preference for her was only observed in the belief‐based accident condition. When it was in common ground that the transgressions were accidents (i.e., from the transgressor's gasp and shocked expression), as in the intent‐based accident condition, 5‐year‐olds recognized that no further explanation was needed (Köymen et al., 2016).

Another interpretation for the transgressor's shocked expression could be that she did not expect her behavior to be observed by anyone, making her expression not about intentions but about her company. We can preclude this interpretation, however, for two reasons. First, the victim was always present and never left the transgressor's side (Appendix A). Second, the transgressor's surprise was linked back to her actions by the experimenter in his narration of the story (e.g., “[Lisa gasps] Lisa ripped the picture. But she did not want to rip the picture. She did it by accident.”). These facial expressions were thus likely perceived by children as pertaining to the transgressor's conduct rather than to her surroundings. Future studies, however, might benefit from including additional comprehension checks to discriminate between these competing explanations.

While children preferred the reason‐giving transgressor more often in the belief‐based accident condition than in the intent‐based accident condition, this difference was not significant for the help and play partner‐choice questions. These questions were perhaps too distinct from the stories depicting the transgressors. Preschoolers have been shown to limit their negative evaluations about a transgressor to the specific transgression caused and may not extend these to novel activities (Oostenbroek & Vaish, 2019).

Across both conditions, 4‐year‐olds' preferences were similar for both the reason‐giving and apology‐only transgressor. This finding is consistent with previous research which suggests young children tend to privilege intentions above beliefs (e.g., Killen et al., 2011). Before 5, then, children may not understand that belief‐based transgressions are indeed accidents and therefore may not value reasons to this effect.

Our exploratory analysis on children's justifications for their preferences showed 5‐year‐olds gave more reason‐related justifications (e.g., “She thought it was hers”) in the belief‐based accident condition (where the reason‐giving transgressor was favored) than in the intent‐based accident condition (where no preference was found). This finding is also consistent with prior research showing young children are more likely to give reasons when solving problems with a clearly correct solution than for problems with multiple, equally plausible answers (Köymen et al., 2020).

What this study shows is that older preschoolers recognize which mistakes need or benefit from more than an apology, and which do not. What Study 1 leaves open, however, is how 5‐year‐olds compare apologies following belief‐based accidents to those following intentional and accidental harm (in which both the act and outcome are matched), and whether the kind of reason accompanying the apology affects their evaluations of the transgressor.

## STUDY 2

In Study 2, the procedure followed that of Study 1, but with the transgressions paired differently. In the intentional condition, children witnessed two seemingly intentional transgressions. One transgressor gave a “good” reason which signaled an unintended outcome (“I'm sorry, I thought this was my picture”) [henceforth “belief‐based transgressor”], the other gave a “bad” reason which made the act and outcome intended (“I'm sorry, I thought your picture wasn't good”) [henceforth “intentional transgressor”]. In the unintentional condition, children saw the belief‐based transgressor compared to a transgressor who caused an intent‐based accident and gave a “good” reason which made the act and outcome unintended (“I'm sorry, I was trying to see the picture better”) [henceforth “accidental transgressor”]. Using revised partner‐choice questions which matched the context of the transgressions, children then selected which transgressor to help with some drawing, to draw some pictures with, and to trust with their picture.

As part of our confirmatory hypotheses, we predicted that in the intentional condition, 5‐year‐olds would prefer the belief‐based transgressor more than the intentional transgressor. We also expected children would show no preference between the transgressors in the unintentional condition, since neither intended the outcome (Proft & Rakoczy, 2019). As part of our exploratory hypotheses, we investigated whether there was a condition difference in the way children justified their preferences.

### Method

The procedure, hypotheses, sample size, exclusion criteria, and statistical analyses were preregistered (https://osf.io/mzcv4).

#### Participants

Forty‐eight 5‐year‐olds (*M* = 5;6, range = 5;1–6;0: 25 girls), who did not participate in Study 1, were randomly assigned to one of two conditions. Data collection took place between April 2021 and November 2021. The mean age of participants did not differ between the intentional (*M* = 5;6, SD = 0;2) and unintentional (*M* = 5;5, SD = 0;3) conditions (*t*(46) = 1.57, *p* = .124, *d* = 0.45). One additional 5‐year‐old was excluded for not answering any of the partner‐choice questions. Children who were native speakers of English were recruited from a database covering northwest England. We did not collect individual data about participants' socioeconomic or ethnic background, but families in this database come from predominantly White, middle‐class backgrounds. Informed parental consent and verbal child assent were obtained before participation in the study.

#### Materials

The stimuli were the same as Study 1, with two exceptions. Firstly, the stimuli were rearranged to compare the different types of transgression. Secondly, verbal cues of intent (i.e., the transgressor's gasp/laugh) were removed to reduce the difference in intentionality between both kinds of accident in the unintentional condition (Appendix B).

#### Procedure

The procedure was identical to that of Study 1, except for the pairing of the transgressions. In the intentional condition, children observed the transgressors cause the same seemingly intentional transgression. The belief‐based transgressor gave a “good” reason which signaled an unintended outcome (“I'm sorry, I thought this was my picture”), whereas the intentional transgressor gave a “bad” reason which made the act and outcome appear intended (“I'm sorry, I thought your picture wasn't good”). In the unintentional condition, children witnessed the belief‐based transgressor and the accidental transgressor apologize and give “good” reasons for their transgressions. The belief‐based transgressor said: “I'm sorry, I thought this was my picture.” The accidental transgressor said: “I'm sorry, I was trying to see the picture better.”

Children were then presented with the revised partner‐choice questions. In the help question, the transgressors were missing crayons needed for drawing pictures. Children were told: “They both ripped up Tom's pictures, but who would you like to give the crayon to? Lisa or Poppy?” In the play question, children chose which transgressor they would rather do some coloring with. In the trust question, a picture (said to be the participant's) sat between the transgressors and children decided which should look after it. Finally, children were asked to justify their preferences.

#### Coding

Coding was the same as that of Study 1. A second coder, blind to predictions, coded 25% of the data (12 children). Agreement was *κ* = 1.00.

### Results

As part of the preliminary analyses, we analyzed whether children's performance in the warm‐up trials (in which children identified the “nicer” character) varied by condition. The mean number of trials answered correctly was 3.79 in the intentional condition and 3.92 in the unintentional condition (out of four trials). Children's warm‐up performance did not differ between conditions (*t*(46) = 1.22, *p* = .229, *d* = 0.35).

**FIGURE 4:**
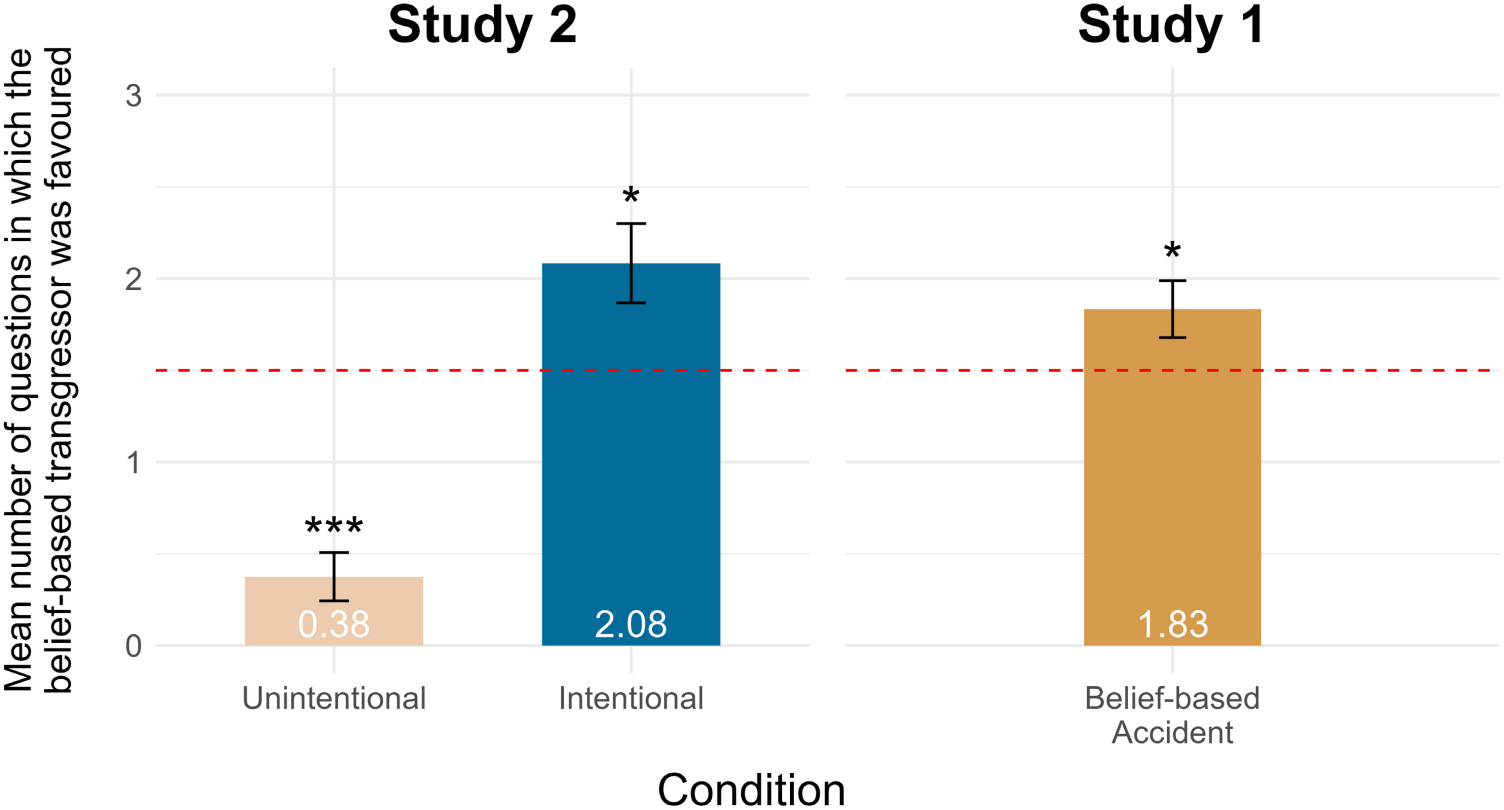
Mean number of partner‐choice questions in which 5‐year‐olds favored the belief‐based transgressor by condition. Five‐year‐olds' performance in the belief‐based accident condition of Study 1 is included for comparison purposes. The red line represents chance. Error bars show standard error. *p < .05, ***p < .001 compared to chance.

We then ran the same four sets of analyses as in Study 1. First, one‐sample t‐tests suggested that 5‐year‐olds showed a significant preference for the belief‐based transgressor in the intentional condition (*t*(23) = 2.70, *p* = .013, *d* = 0.55), and a significant preference for the accidental transgressor in the unintentional condition (*t*(23) = 8.52, *p <* .001, *d* = 1.74; Figure 4).

Second, to ascertain whether children's overall preference for the belief‐based transgressor differed across conditions, we ran a between‐subjects analysis of variance (ANOVA). A significant main effect of condition (*F*(1, 45) = 47.00, *p <* .001, $\eta_{p}^{2}$ = .511; see Supplementary Materials E for the output summary) showed 5‐year‐olds favored the belief‐based transgressor significantly more often in the intentional condition than in the unintentional condition (Figure 4). To explore whether the performance of 5‐year‐olds varied when controlling for age, a between‐subjects ANCOVA in which age was treated as a continuous variable (i.e., measured in months) was also conducted. The interaction between age (in months) and condition was not significant (*F*(1, 43) = 1.09, *p* = .301, $\eta_{p}^{2}$ = .025). The only significant main effect was that of gender (*F*(1, 43) = 4.19, *p* = .047, $\eta_{p}^{2}$ = .089; see Supplementary Materials F for the output summary). When controlling for age, girls favored the belief‐based transgressor more often than boys.

**FIGURE 5:**
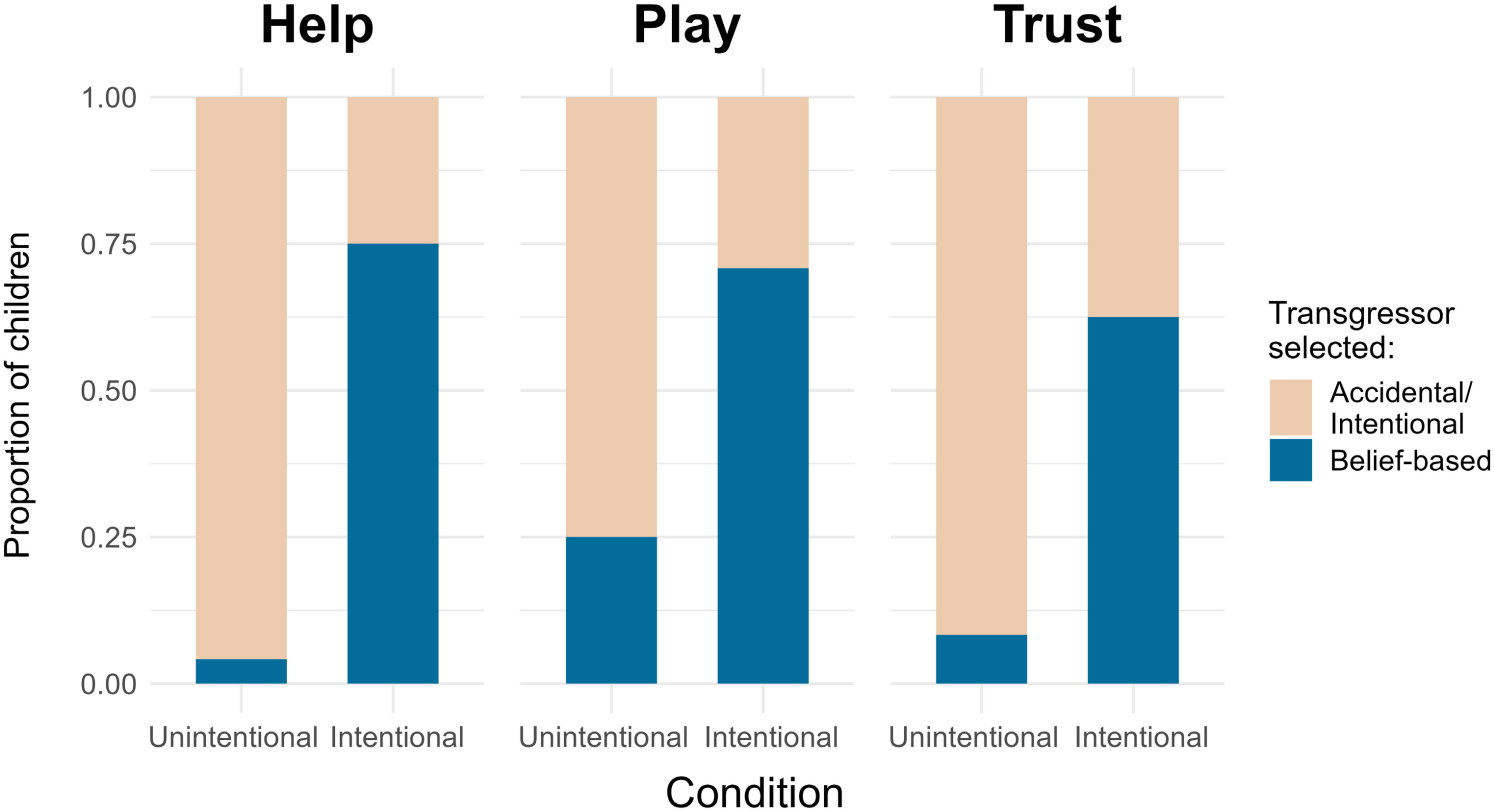
Five‐year‐old children's preferences in Study 2 for the belief‐based transgressor across the help, play and trust questions by condition.

Third, to determine children's preferences for the belief‐based transgressor across each partner‐choice question, we ran three GLMs with binomial error distribution (see Supplementary Materials G for the model summaries). The response variable was the binary measure of whether the child chose the belief‐based transgressor. The full model included condition and gender. The null model included gender only. Each analysis revealed the same pattern. Five‐year‐olds were more likely to help (*χ* ^2^ = 29.07, df = 1, *p* < .001), play with (*χ* ^2^ = 11.32, df = 1, *p* = .001), and trust (*χ* ^2^ = 18.44, df = 1, *p* < .001) the belief‐based transgressor in the intentional condition than in the unintentional condition (Figure 5).

Finally, to investigate children's justifications for their preferences, we fitted a GLM with binomial error distribution (see Supplementary Materials H for the model summary). The response variable was the binary measure of whether children produced a reason‐related justification. The models were the same as the previous GLMs. Five‐year‐olds gave significantly more reason‐related justifications in the intentional condition than in the unintentional condition (*χ* ^2^ = 8.62, df = 1, *p* = .003; Figure 3).

### Discussion

When both transgressions appeared intentional, 5‐year‐olds preferred to help, play with, and trust the transgressor who gave a “good” reason explaining that the outcome was unintended over the transgressor who gave a “bad” reason, which made both the act and outcome seem deliberate. Thus, when intentional harm was perceived, children attended to the reasons provided and trusted the transgressor with the better reason.

Conversely, in the unintentional condition wherein the belief‐based accident was pitted against an intent‐based one, 5‐year‐olds showed a preference for the accidental transgressor and did not treat both kinds of accident equivalently. Presumably, children perceived the intent‐based accident as being “more” of an accident. This will be discussed further in the General Discussion.

Despite having clear preferences in each condition, 5‐year‐olds provided more reason‐related justifications in the intentional condition than in the unintentional condition. This condition difference might indicate that the “correct” decision was obvious in the intentional condition (Köymen et al., 2020). In the unintentional condition, children had a harder time justifying their preference for the accidental transgressor, presumably because they recognized that the belief‐based transgressor had a similarly “good” reason for her transgression.

In our exploratory analysis, we observed gender differences when controlling for age. In comparison to boys, girls had a greater overall preference for the belief‐based transgressor which might indicate a heightened awareness for others' mistaken beliefs. Previous studies have found a slight female advantage on false belief tasks (Charman et al., 2002), maybe because girls are recipients of more mental state talk than boys (e.g., Leaper et al., 1998).

## GENERAL DISCUSSION

Giving reasons for belief‐based accidents pays dividends, as it reinterprets others' perception of the event. In Study 1, we showed that 5‐year‐olds, but not 4‐year‐olds, understand when reasons should be given for an accident, recognizing that explaining belief‐based accidents is more important than for intent‐based ones. In Study 2, we also showed that the reason accompanying the apology changed children's interpretation of the same seemingly intentional transgression. The “good” reason indicated that the transgressor had unknowingly caused harm which helped to mitigate her transgression, whereas the “bad” reason had the reverse effect. Taken together, these results suggest 5‐year‐olds understand when and what kinds of reasons should accompany apologies.

Previous research has found that 5‐year‐olds recognize “good” reasons for moral transgressions (Kanngiesser et al., 2021; Mammen et al., 2018, 2021). Here, we extend these findings by showing that 5‐year‐olds also recognize *when* reasons ought to be given. Simply apologizing for a belief‐based accident was perceived to be almost as bad as giving a poor reason (Figure 4). Further, 5‐year‐olds often explicitly referred to the belief‐based transgressor's reason (e.g., “She thought it was hers”) as being the basis for their preferences. Thus, children this age already reason about reasons and engage in so‐called “meta‐talk” (Hartwell et al., 2022; Köymen & Engelmann, 2022; Köymen & Tomasello, 2018, 2020).

Our findings might also have implications for the development of mental state reasoning in children. Before age 7, children often find it challenging to identify others' false beliefs when these beliefs have moral implications (e.g., Killen et al., 2011). Although our study was not a test of children's morally‐relevant theory of mind per se, our findings suggest that 5‐year‐olds were aware of the transgressor's mental state and used this information in their normative judgments (e.g., “She thought it was hers”). Unlike in earlier work (Proft & Rakoczy, 2019), however, 5‐year‐olds in the present studies made these belief‐based judgments without scaffolding. Their unaided success might be explained by the way in which the false belief was presented. Previously, the transgressor's mistaken belief was experienced by children first‐hand (Amir et al., 2021) or was introduced as part of the narrative told by the experimenter (Proft & Rakoczy, 2019). Here, participants were uninvolved observers who inferred it from the reasons provided by the transgressor herself. Four‐year‐olds, conversely, consistently performed at chance. Despite their success in traditional false belief tasks at this age (Wellman et al., 2001), children's sensitivity to *morally‐relevant* false beliefs seemingly develops from age 5 onward.

Online testing has been increasingly implemented since the COVID‐19 pandemic. This change in research methodology has raised questions around participant engagement and data quality. Across both studies, we implemented a procedure in which the experimenter shared and actively moderated the test material via video‐conferencing. This interactive approach to remote testing has been shown to produce similar outcomes (e.g., participant responsiveness and data quality) to those observed in lab‐based experiments (Schidelko et al., 2021), and this conclusion rings true from our own experiences of piloting Study 1 in the lab. Although online data collection poses a number of challenges (Kominsky et al., 2021), moderated testing paradigms are a promising alternative to traditional in‐lab test settings.

A potential concern across the current studies was our use of a partner‐choice paradigm. Although we wanted to reduce the demands placed on children and show that they display a systematic preference within this constrained format, these forced‐choice questions obliged children to select a transgressor when participants might have preferred to choose both or neither. This might explain why 5‐year‐olds in Study 2 did not evaluate intent‐based and belief‐based accidents equivalently. This is not to say that 5‐year‐olds do not understand belief‐based mistakes (as they favored the belief‐based transgressor in other conditions), but making children choose one transgressor over another possibly made the intent‐based accident (unintended action‐unintended outcome) appear as “more” of an accident compared to the belief‐based one (intended action‐unintended outcome). Future partner‐choice tasks could compare each transgressor to a neutral character rather than pitting them against each other (see Vaish et al., 2010) to allow children to select both transgressors in turn, or neither.

A further caveat is the generalizability of the present findings. Cross‐cultural studies have established that small‐scale “opacity of mind” societies (e.g., Fiji) prioritize outcomes over intentions when evaluating transgressions (e.g., McNamara et al., 2019). Thus, the developmental patterns described here and in the wider literature may not apply universally. More cross‐cultural research into children's intent‐based and belief‐based judgments is therefore needed.

To conclude, unlike for intent‐based accidents, the present studies show 5‐year‐old children recognize the importance of explaining belief‐based accidents, and explaining them well.

## Supporting information


Appendix S1.

Appendix S2.

## Data Availability

Both studies reported in this paper were preregistered. The preregistration forms are publicly accessible at: https://osf.io/jahzx (Study 1) and https://osf.io/mzcv4 (Study 2). Data, materials and statistical scripts are available from the corresponding author upon request.
