# Supplementary material for: Five‐year‐old children value reasons in apologies for belief‐based accidents
Source: Child Dev. 2023 Jan 24;94(3):e143–53. doi: 10.1111/cdev.13893 (PMC10952182; doi:10.1111/cdev.13893)
Supplement: Supplementary file 1 — Appendix S1. [file CDEV-94-e143-s002.docx]

**Appendix A**

**Study 1: Stimuli and Narration**

| Warm-up: The “nicer” character | | | |
| --- | --- | --- | --- |
| 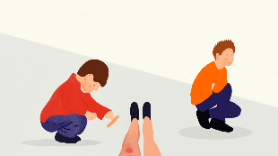 | | 1.1. Some boys were playing outside. One of them fell over and hurt their knee. The boy in red helped his friend. The boy in orange did not.  Which boy do you like more, the one in red or the one in orange? | |
| 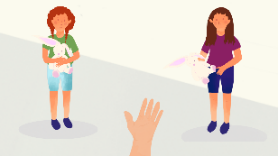 | | 1.2. Some girls were playing with their toys. Their friend also wanted to play. The girl in green did not share with her friend. The girl in purple shared with her friend.  Which girl do you like more, the one in green or the one in purple? | |
| 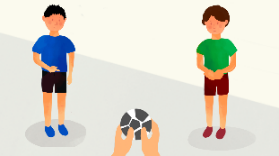 | | 1.3. Both boys wanted to play with the ball. The boy in blue said “Give me that”. The boy in green said “Can I have that, please?”  Which boy do you like more, the one in blue or the one in green? | |
| 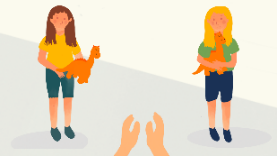 | | 1.4. Both girls were given a new toy. The girl in yellow said “thank you”. The girl in green did not.  Which girl do you like more, the one in yellow or the one in green? | |
| Test trial | | | |
| Intent-based accident condition | | Belief-based accident condition | |
| 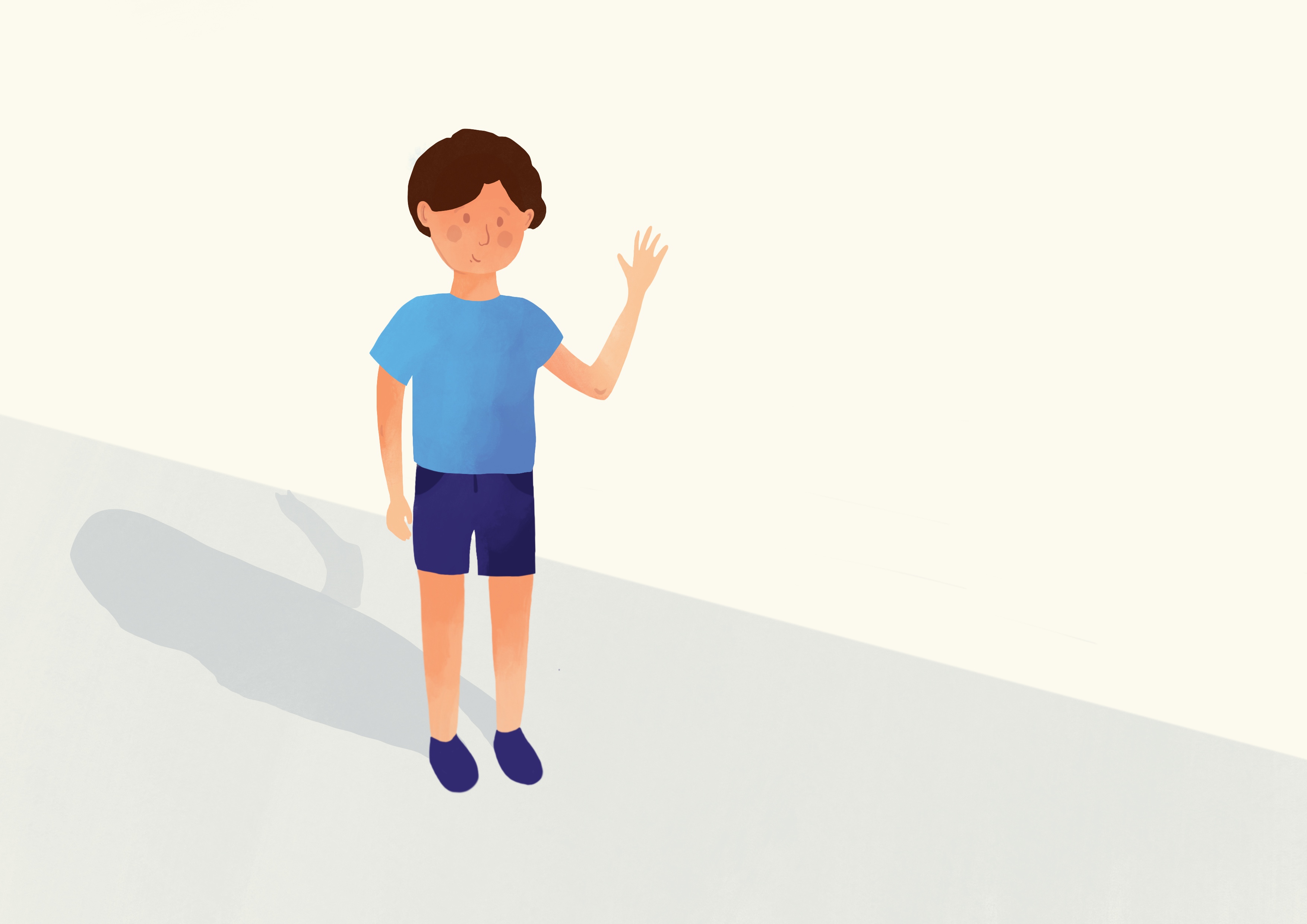 | 2.1. This is Tom. Tom enjoys drawing pictures. | 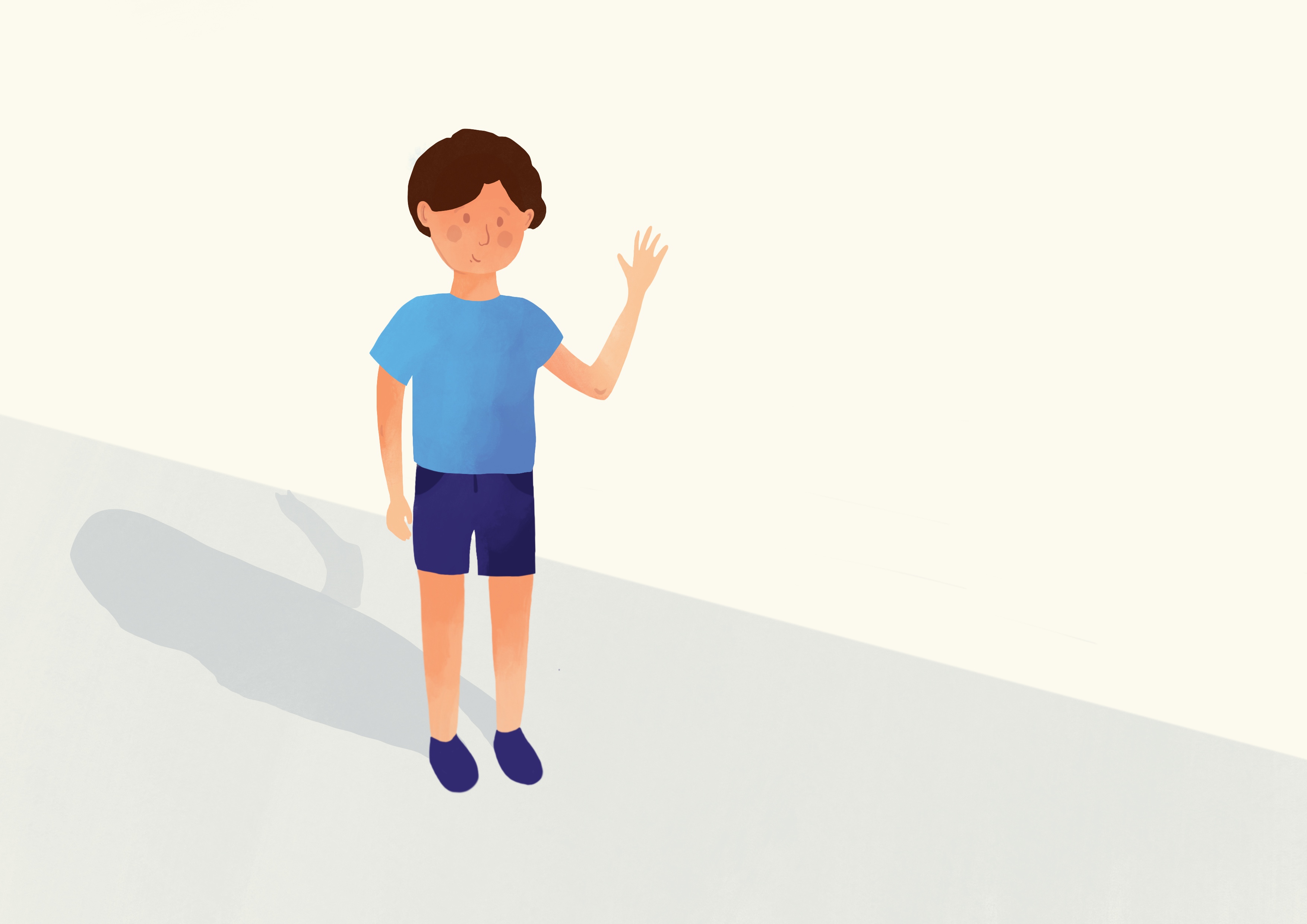 | 2.1. This is Tom. Tom enjoys drawing pictures. |
| 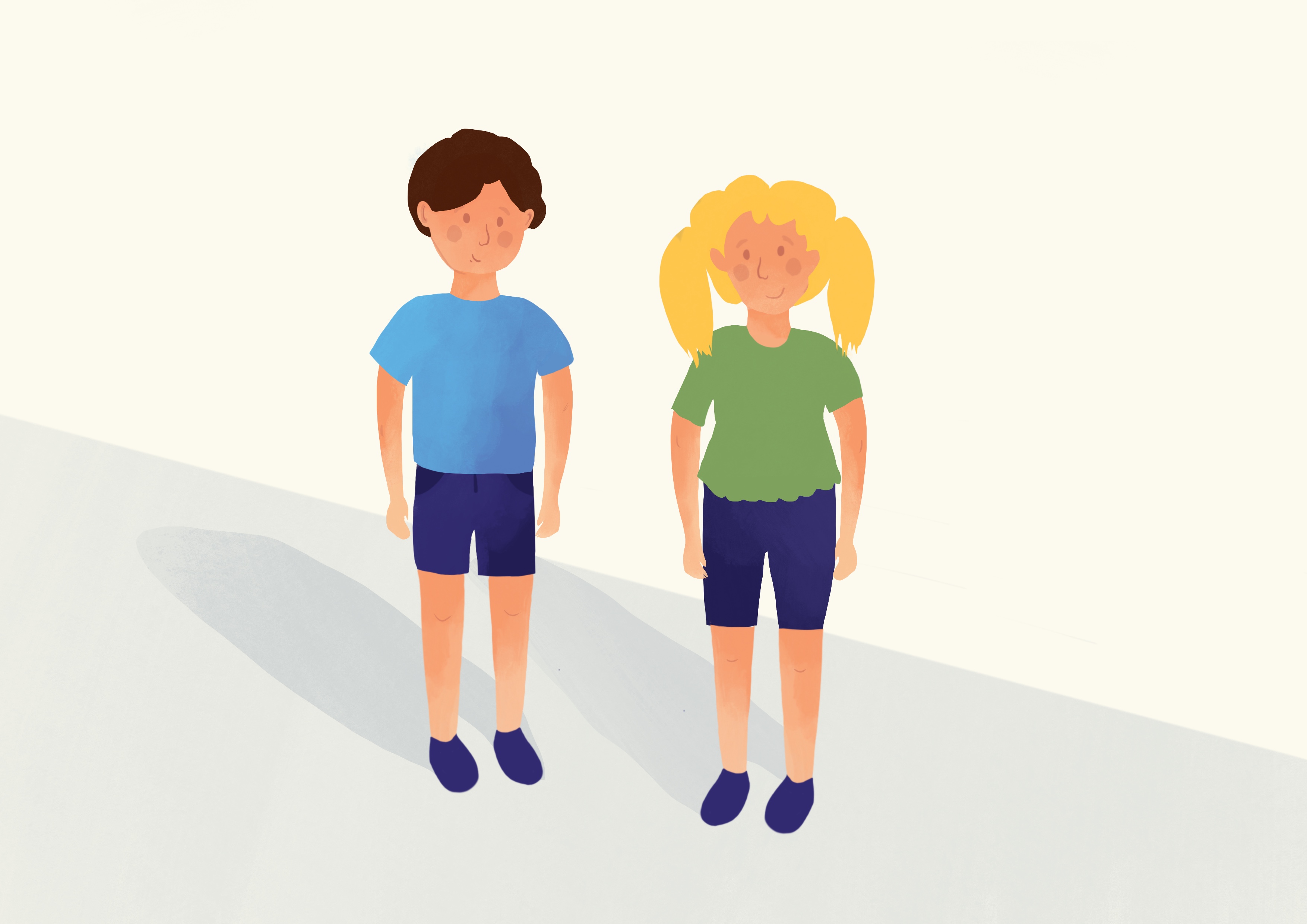 | 2.2. One day, Tom invited his friend Lisa over to play. Tom and Lisa wanted to draw some pictures. | 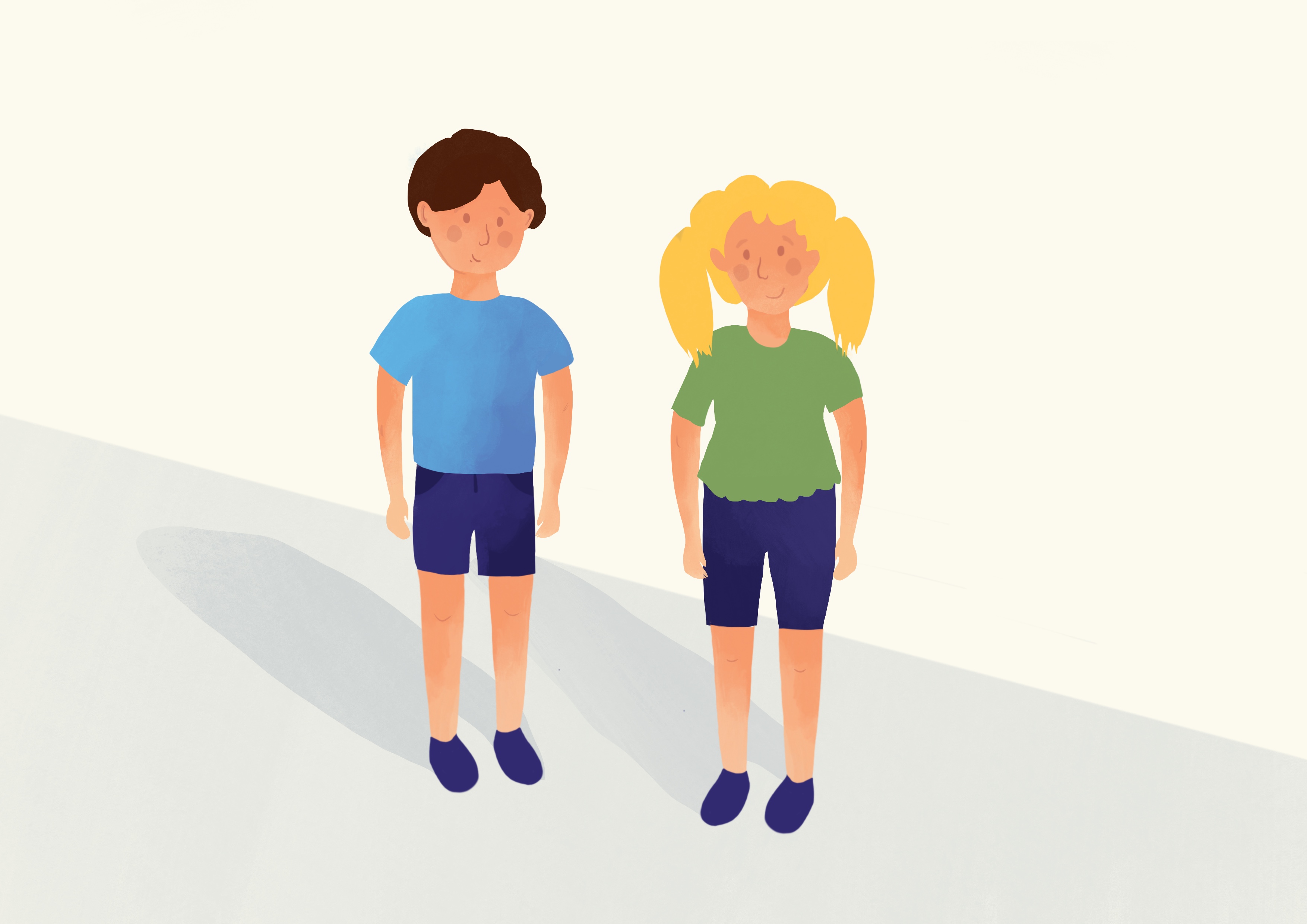 | 2.2. One day, Tom invited his friend Lisa over to play. Tom and Lisa wanted to draw some pictures. |
| 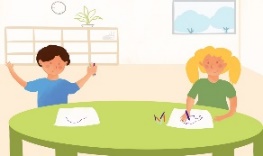 | 2.3. Tom and Lisa sat down to do some drawing. | 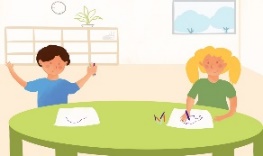 | 2.3. Tom and Lisa sat down to do some drawing. |
| 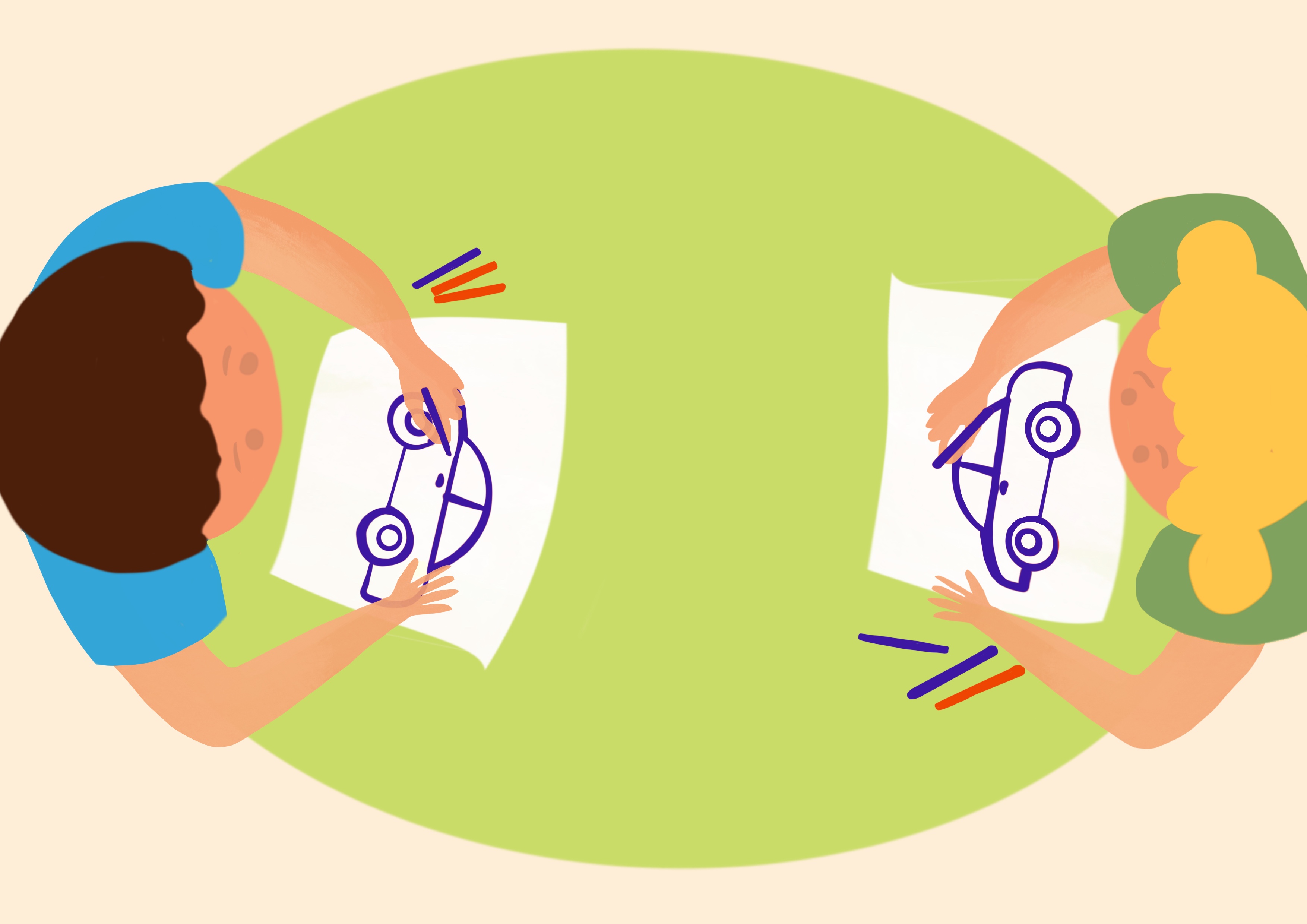 | 2.4. Tom and Lisa decided they wanted to draw cars. Tom and Lisa decided to colour their cars in blue. Tom was very happy with his car. He liked it a lot. | 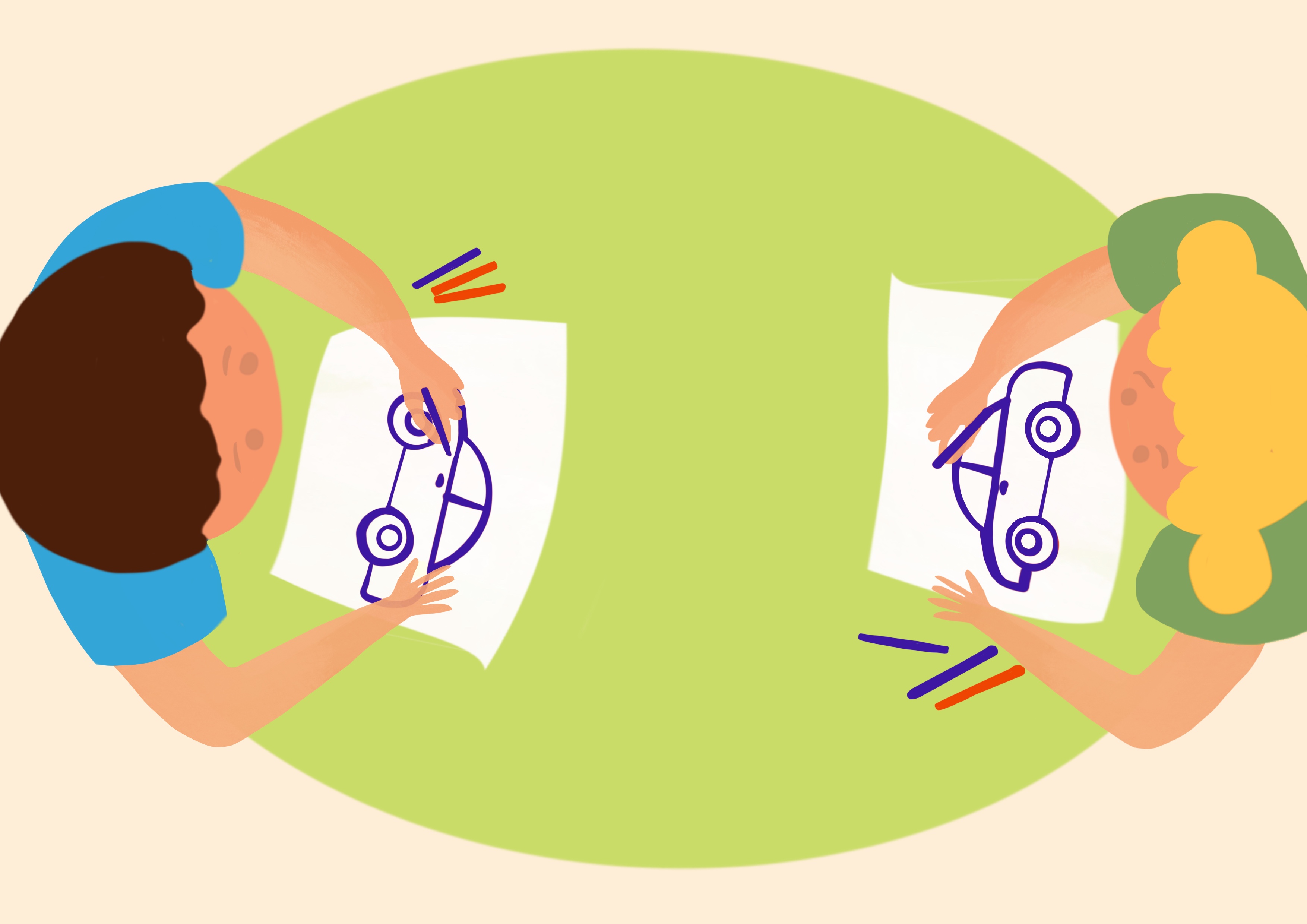 | 2.4. Tom and Lisa decided they wanted to draw cars. Tom and Lisa decided to colour their cars in blue. Tom was very happy with his car. He liked it a lot. |
| 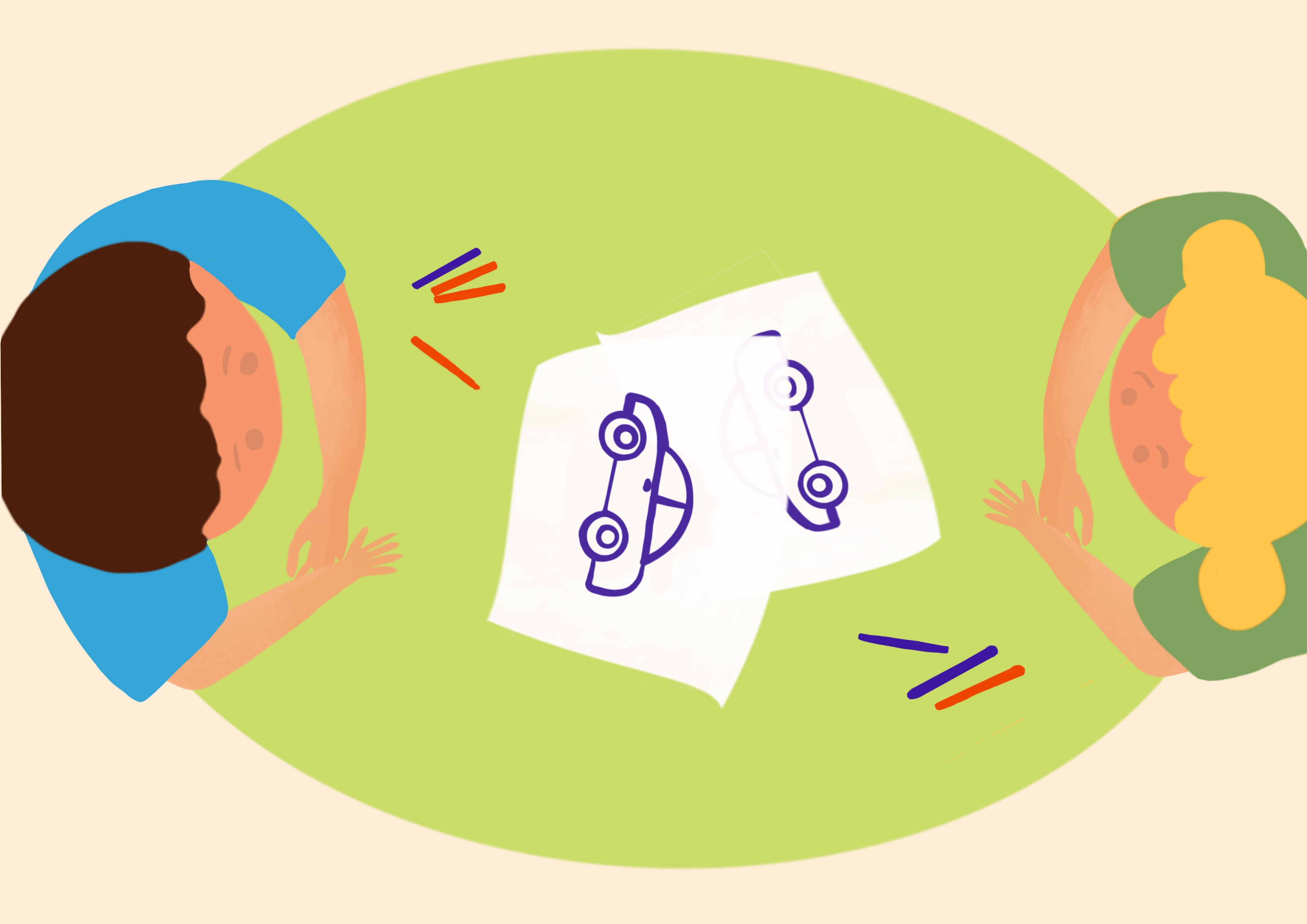 | 2.5. When Tom and Lisa were finished. Tom and Lisa put their pictures in the middle of the table. | 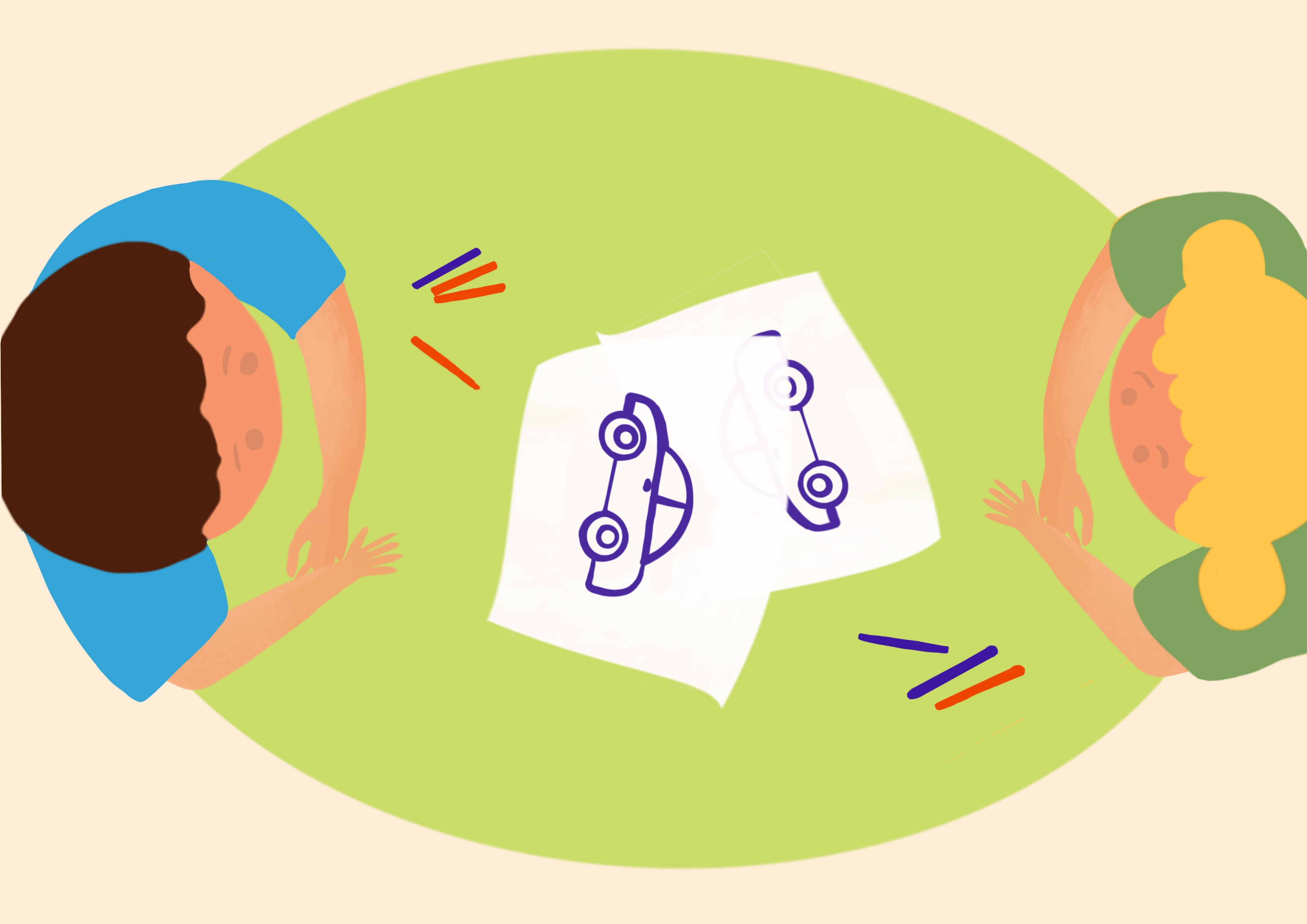 | 2.5. When Tom and Lisa were finished. Tom and Lisa put their pictures in the middle of the table. |
| 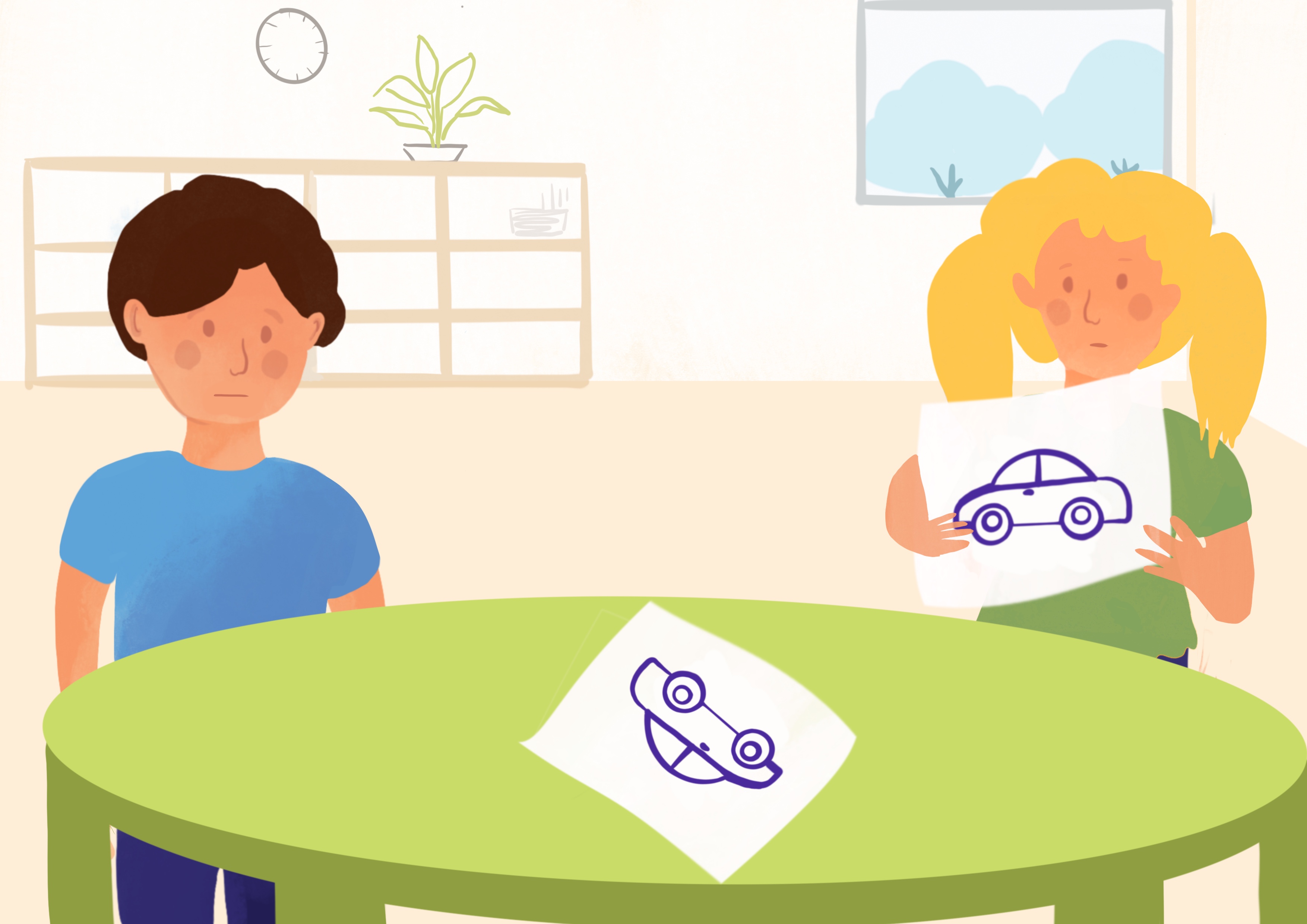 | 2.6. Afterwards, Lisa picked up a picture. | 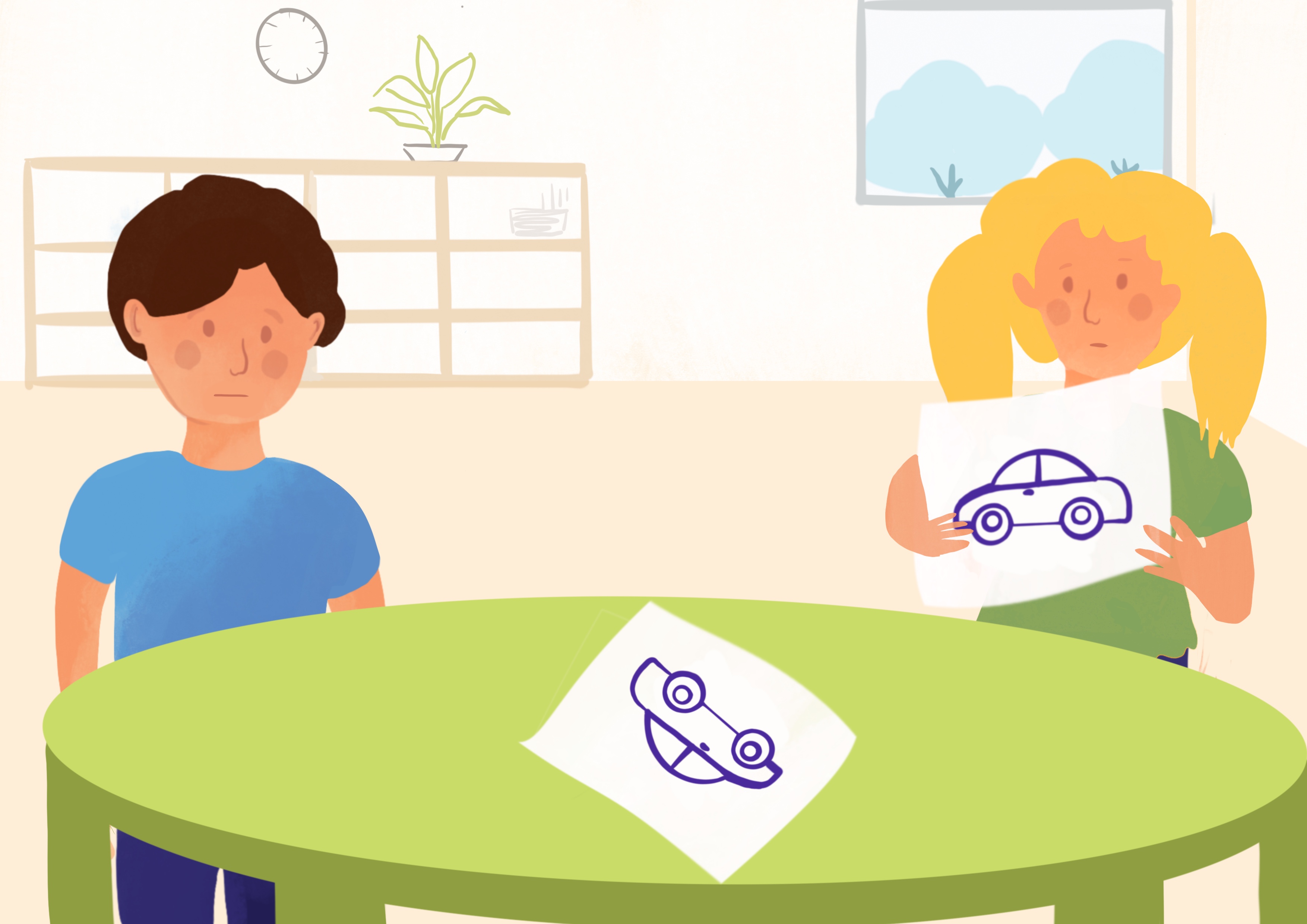 | 2.6. Afterwards, Lisa picked up a picture. |
| 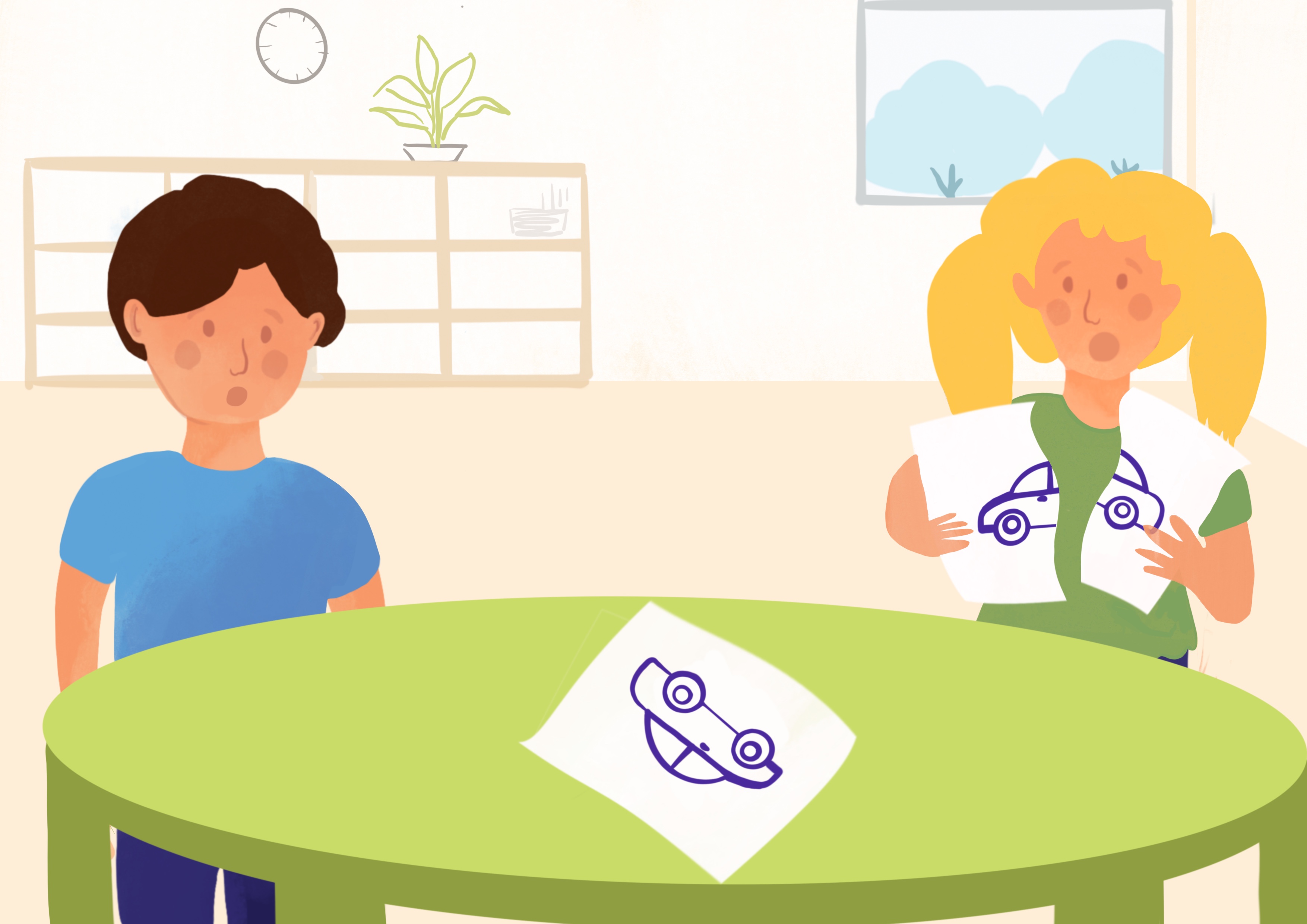 | 2.7. [Lisa gasps] Lisa ripped the picture. But she did not want to rip the picture. She did it by accident. | 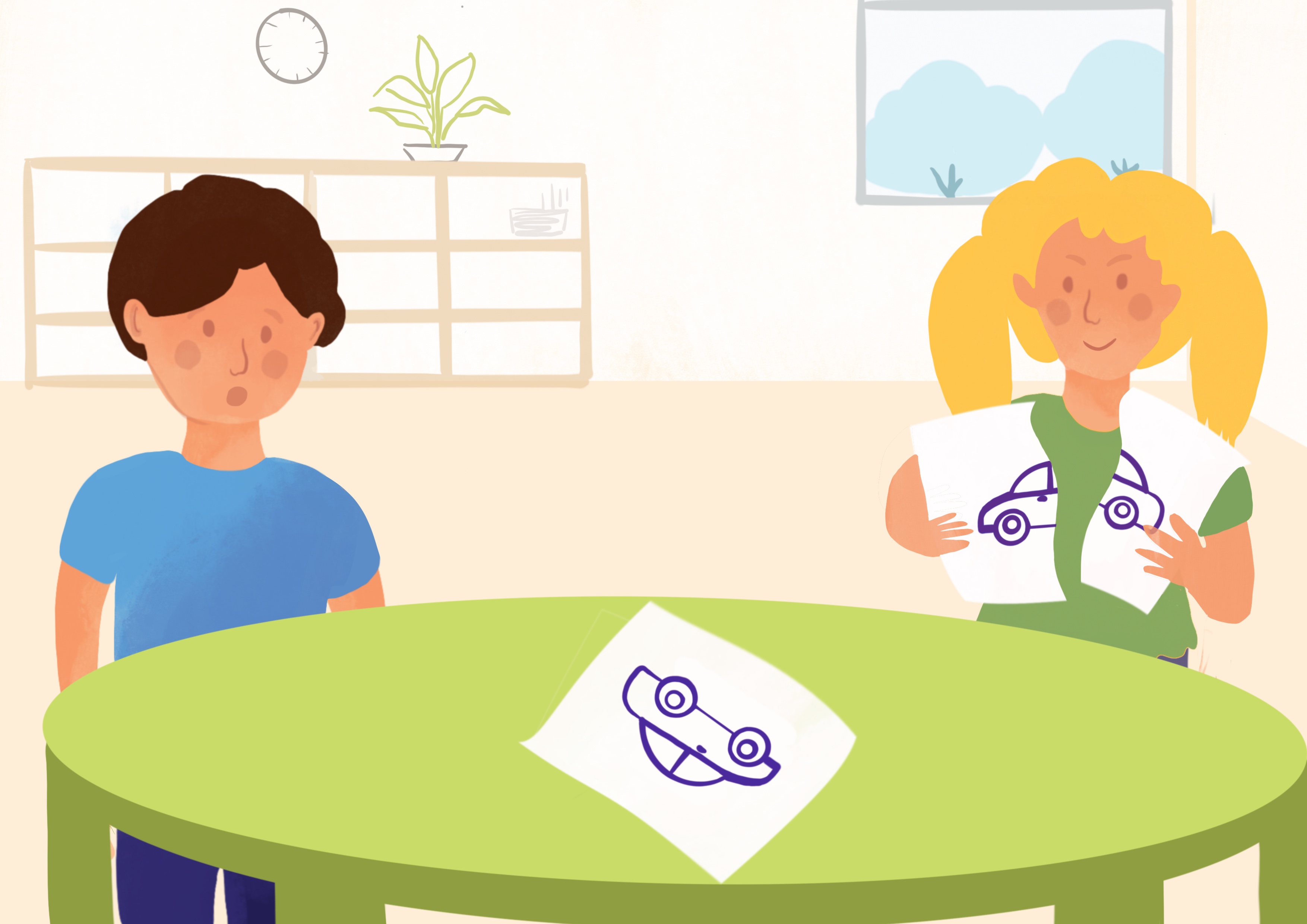 | 2.7. [Lisa laughs] Lisa ripped the picture. She wanted to rip the picture. She did it on purpose. |
| 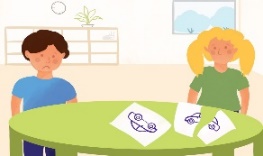 | 2.8. Tom looked at the ripped picture. “That was my picture!” said Tom. ***“I’m sorry”*** said Lisa. Tom felt sad. | 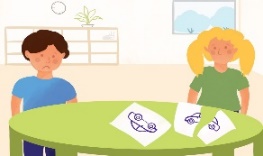 | 2.8. Tom looked at the ripped picture. “That was my picture!” said Tom. ***“I’m sorry”*** said Lisa. Tom felt sad. |
| 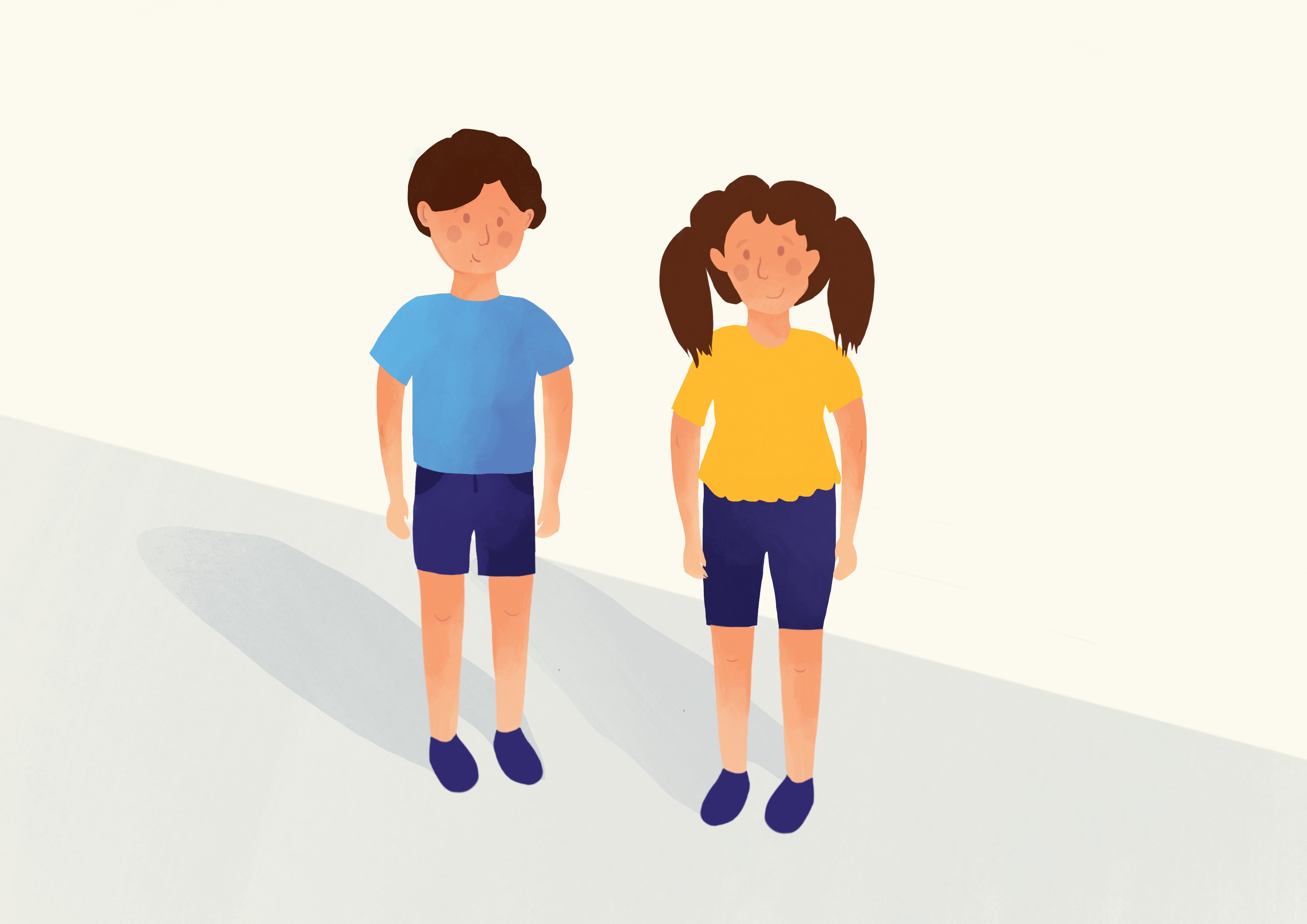 | 2.9. The next day, Tom invited his friend Poppy over to play. Tom and Poppy wanted to draw some pictures. | 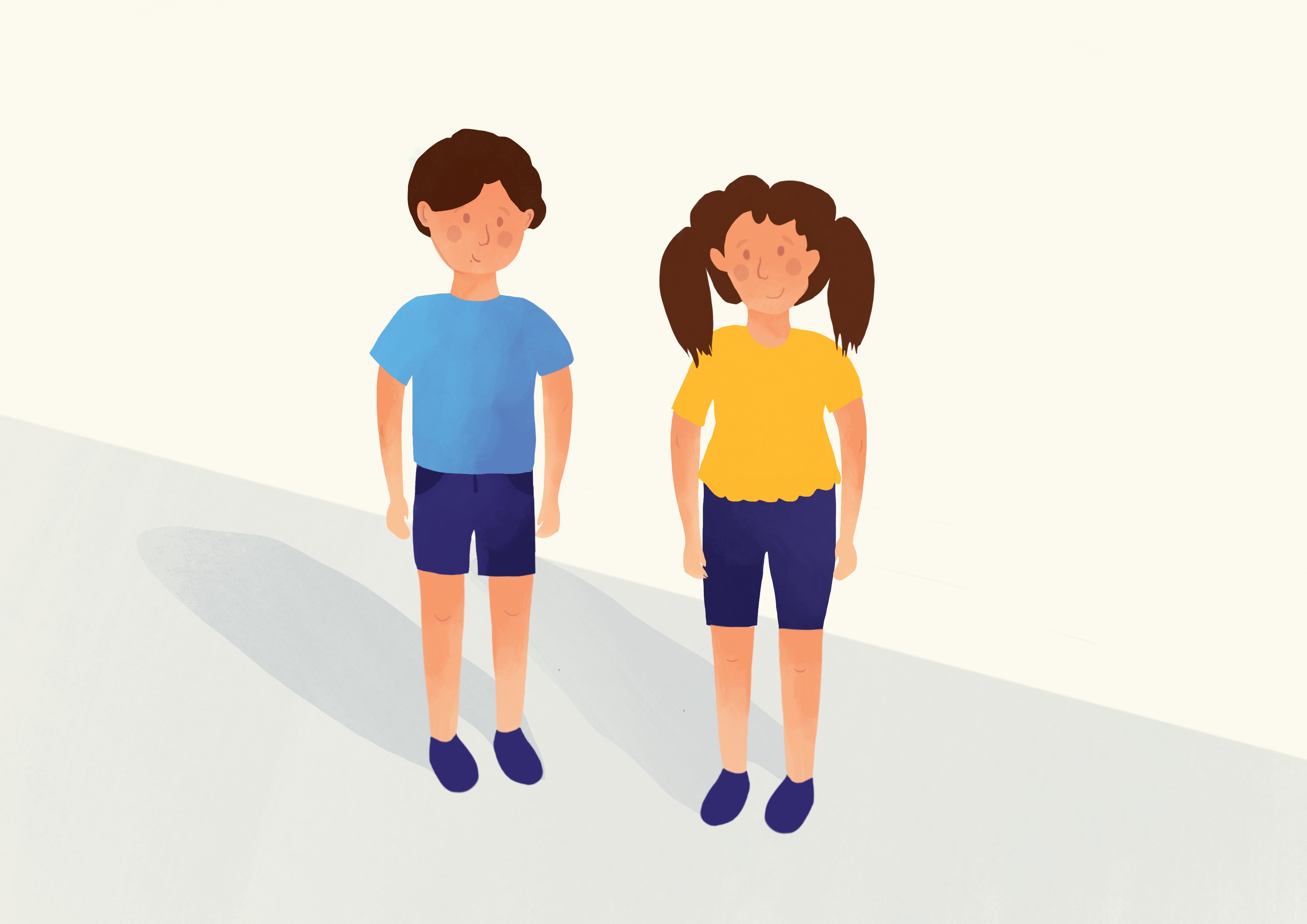 | 2.9. The next day, Tom invited his friend Poppy over to play. Tom and Poppy wanted to draw some pictures. |
| 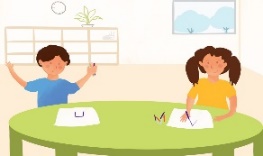 | 2.10. Tom and Poppy sat down to do some drawing. | 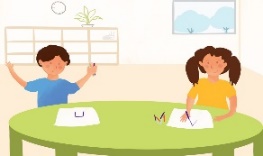 | 2.10. Tom and Poppy sat down to do some drawing. |
| 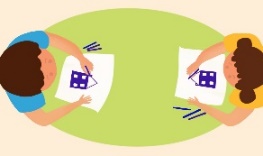 | 2.11. Tom and Poppy decided they wanted to draw houses. Tom and Poppy decided to colour their houses in blue. Tom was very happy with his house. He liked it a lot. | 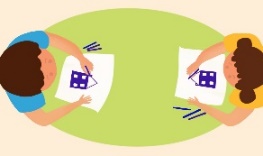 | 2.11. Tom and Poppy decided they wanted to draw houses. Tom and Poppy decided to colour their houses in blue. Tom was very happy with his house. He liked it a lot. |
| 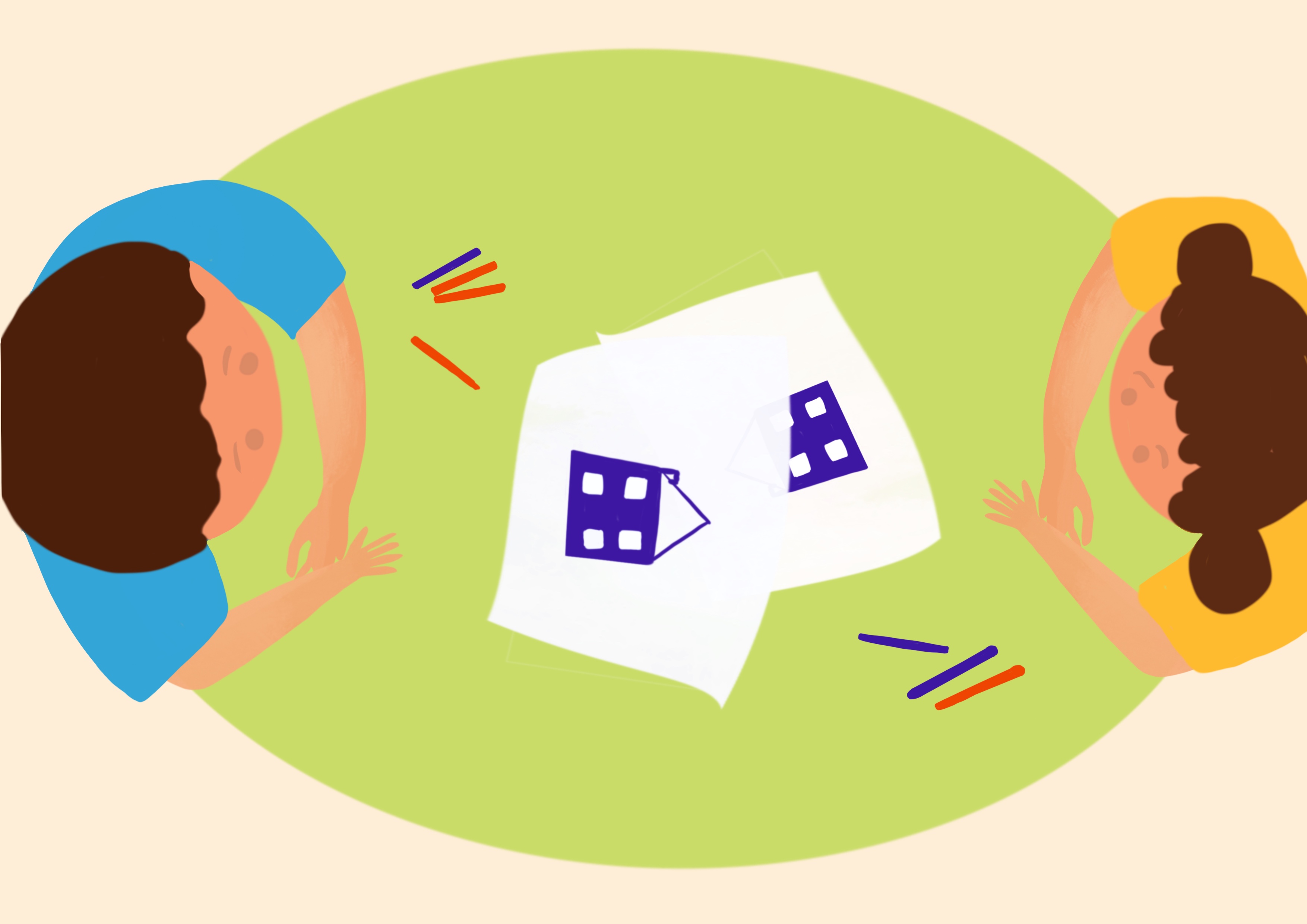 | 2.12. When Tom and Poppy were finished. Tom and Poppy put their pictures in the middle of the table. | 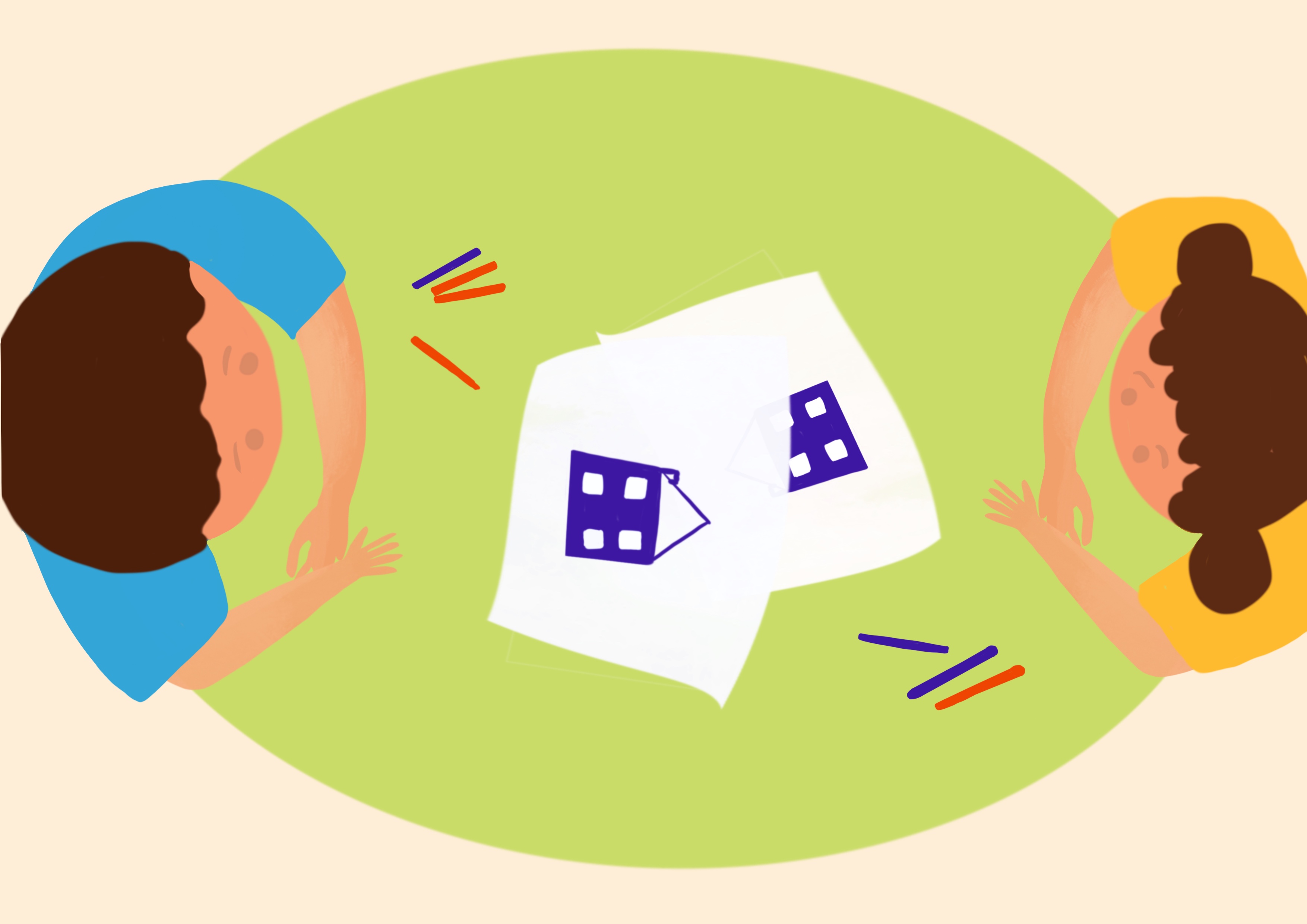 | 2.12. When Tom and Poppy were finished. Tom and Poppy put their pictures in the middle of the table. |
| 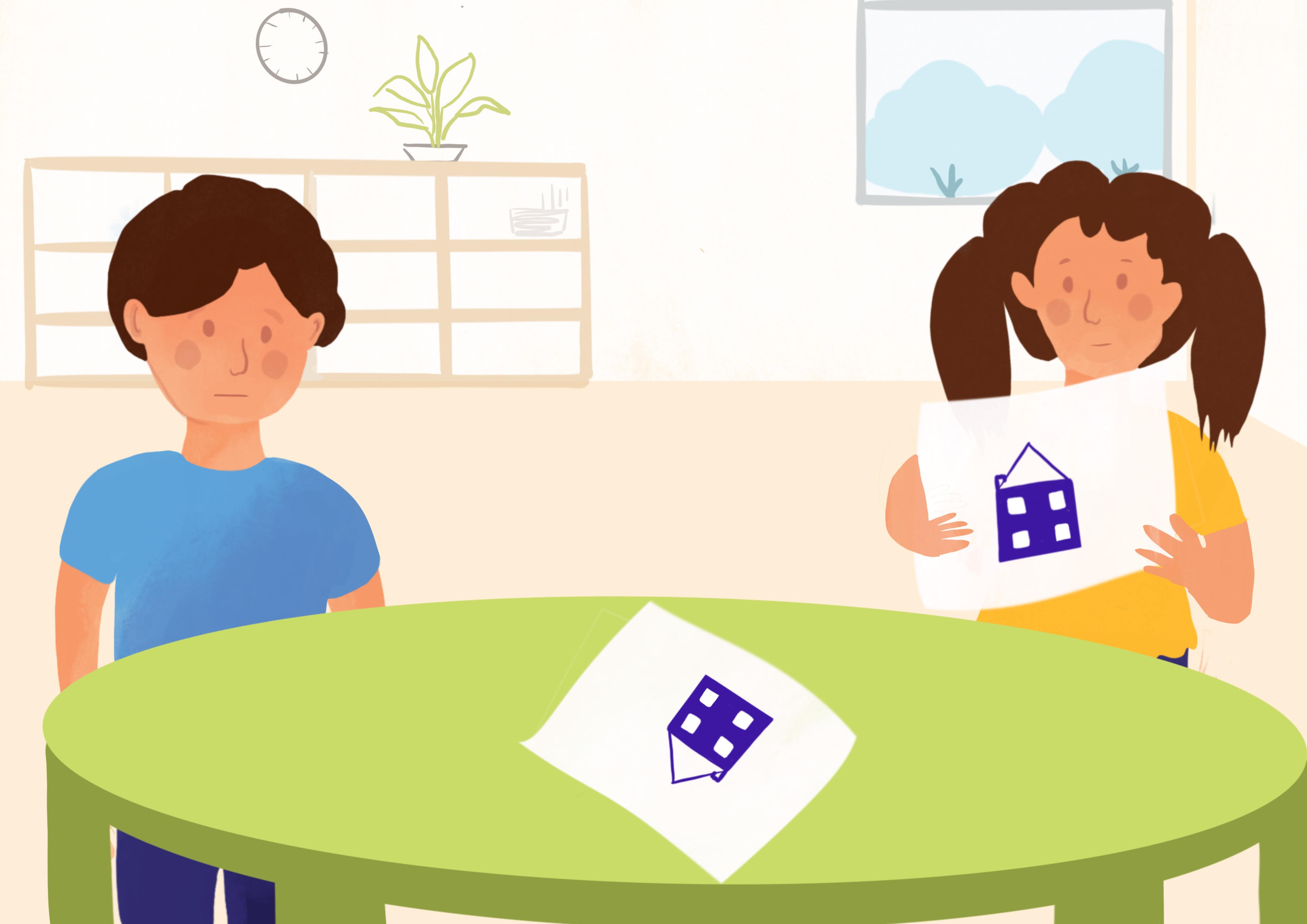 | 2.13. Afterwards, Poppy picked up a picture. | 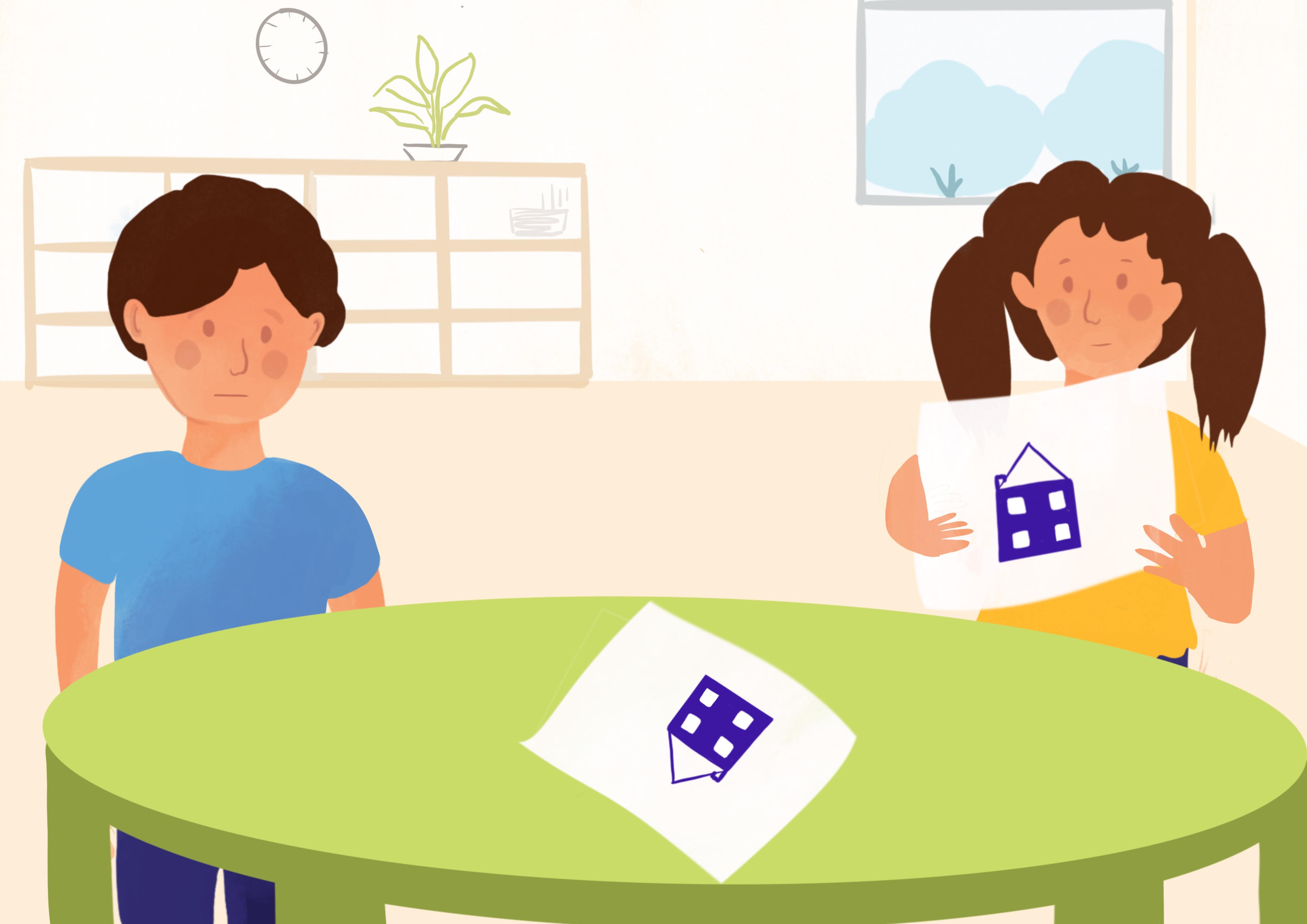 | 2.13. Afterwards, Poppy picked up a picture. |
| 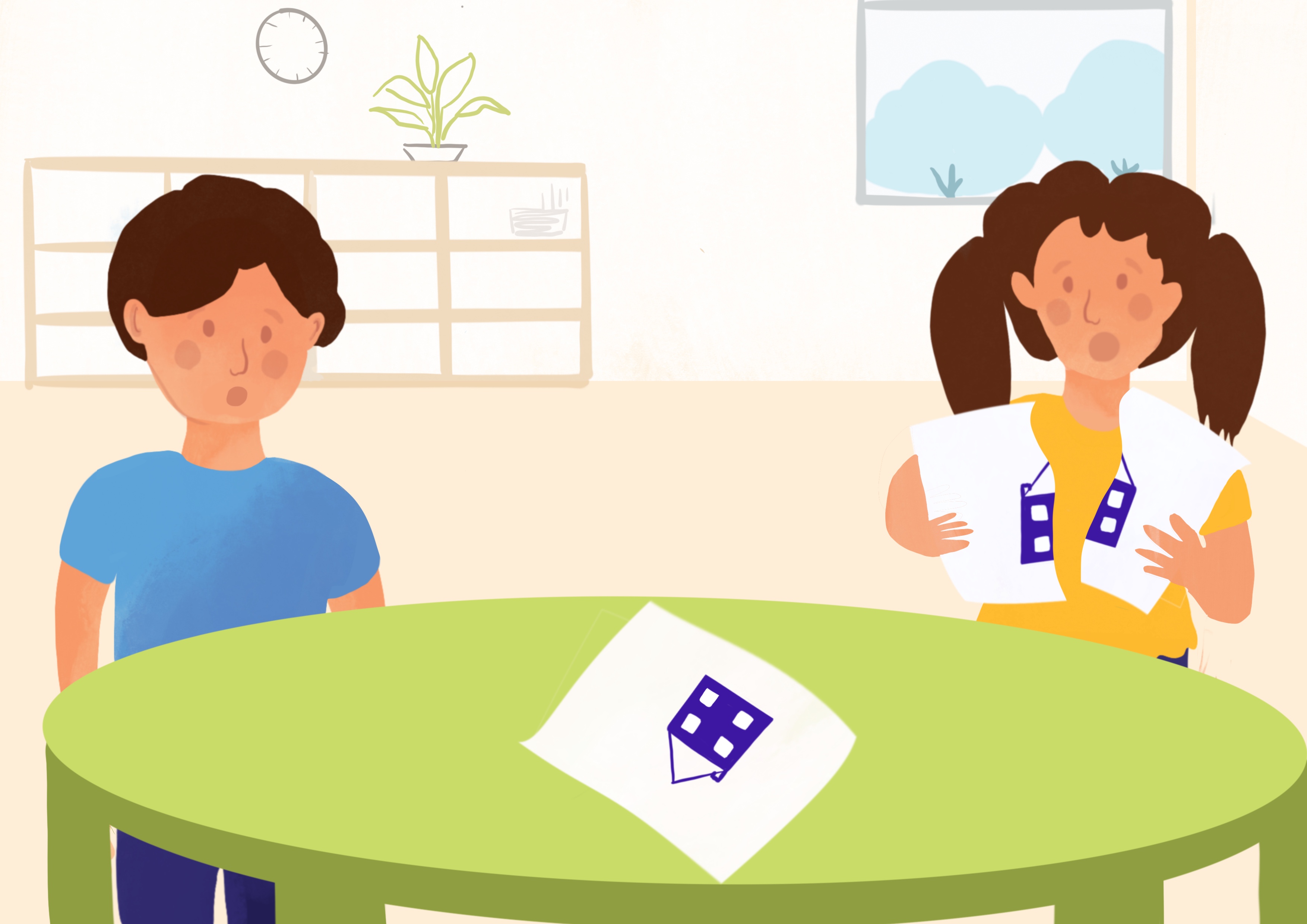 | 2.14. [Poppy gasps] Poppy ripped the picture. But she did not want to rip the picture. She did it by accident. | 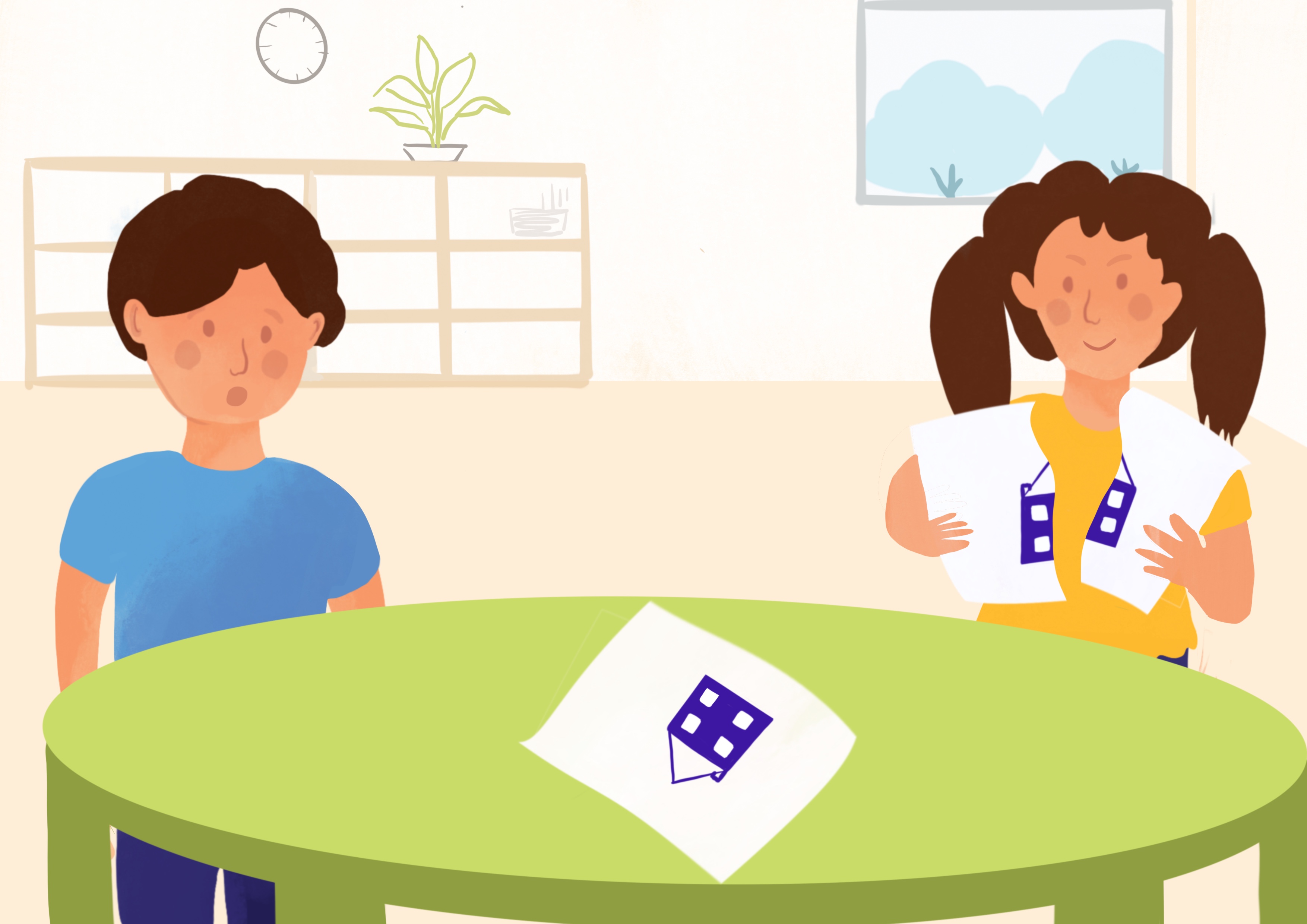 | 2.14. [Poppy laughs] Poppy ripped the picture. She wanted to rip the picture. She did it on purpose. |
| 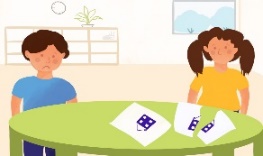 | 2.15. Tom looked at the ripped picture. “That was my picture!” said Tom. ***“I’m sorry,* *I was trying to see the picture better”*** said Poppy. Tom felt sad. | 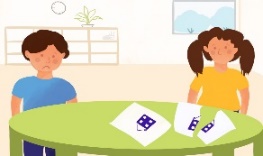 | 2.15. Tom looked at the ripped picture. “That was my picture!” said Tom. ***“I’m sorry,* *I thought this was my picture”*** said Poppy. Tom felt sad. |
| Partner-choice questions | | | |
| 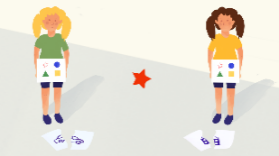 | | 3.1. Both Poppy and Lisa are doing a puzzle. They are both missing the same piece. Here is the piece. You can decide who will get it.  They both ripped up Tom’s pictures, but who would you like to help? Lisa or Poppy? | |
| 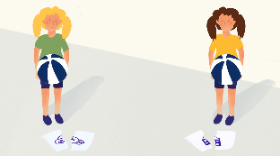 | | 3.2. Both Poppy and Lisa would like to play with you.  They both ripped up Tom’s pictures, but who would you like to play with? Lisa or Poppy? | |
| 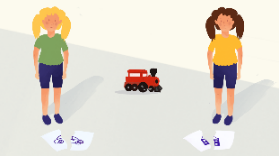 | | 3.3. This is your new toy train. Both Poppy and Lisa would like to look after it for you.  They both ripped up Tom’s pictures, but who would you like to look after your toy? Lisa or Poppy? | |

**Appendix B**

**Study 2: Stimuli and Narration**

| Test trial | | | |
| --- | --- | --- | --- |
| Unintentional condition | | Intentional condition | |
| 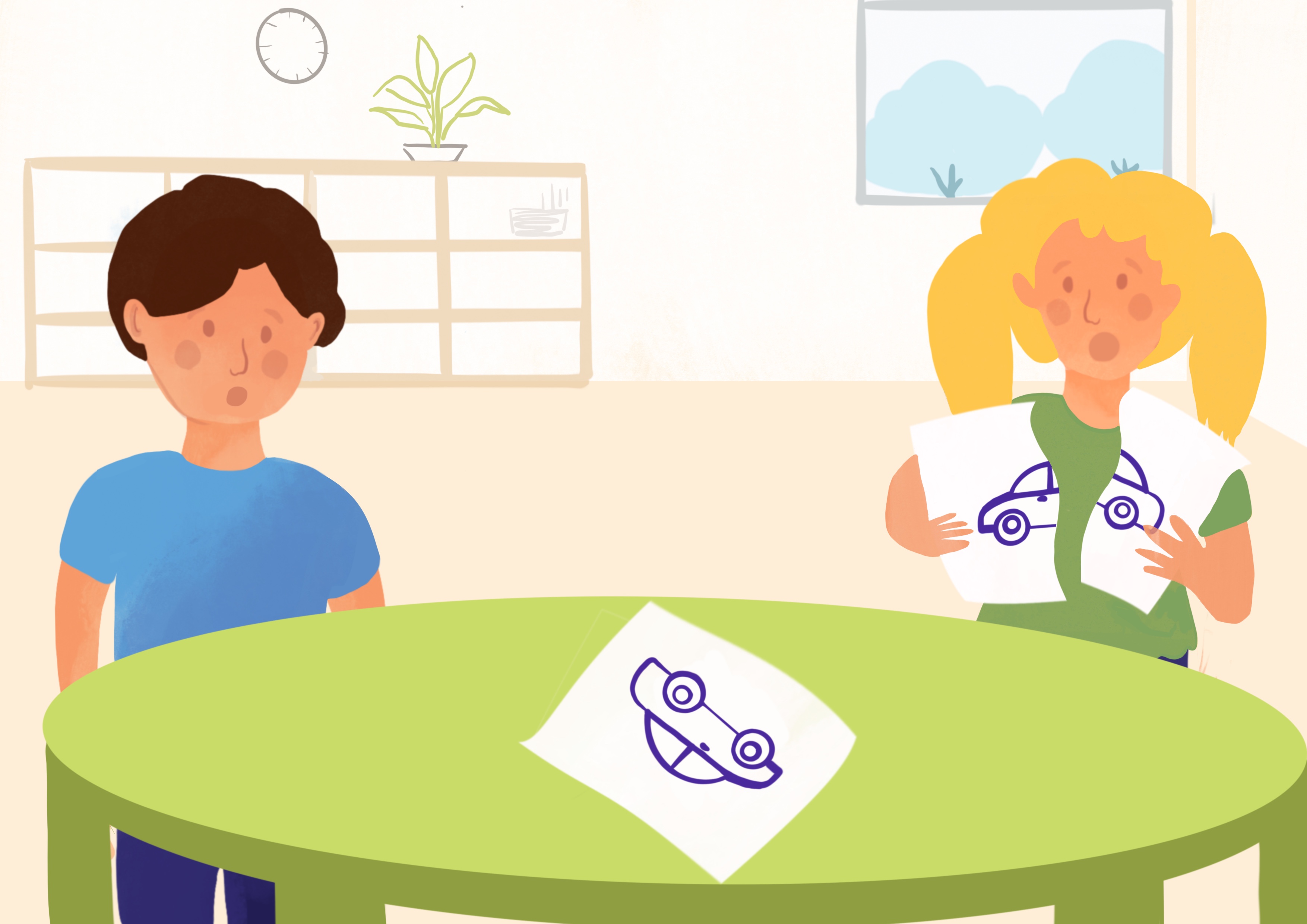 | 2.7. Lisa ripped the picture. But she did not want to rip the picture. She did it by accident. | 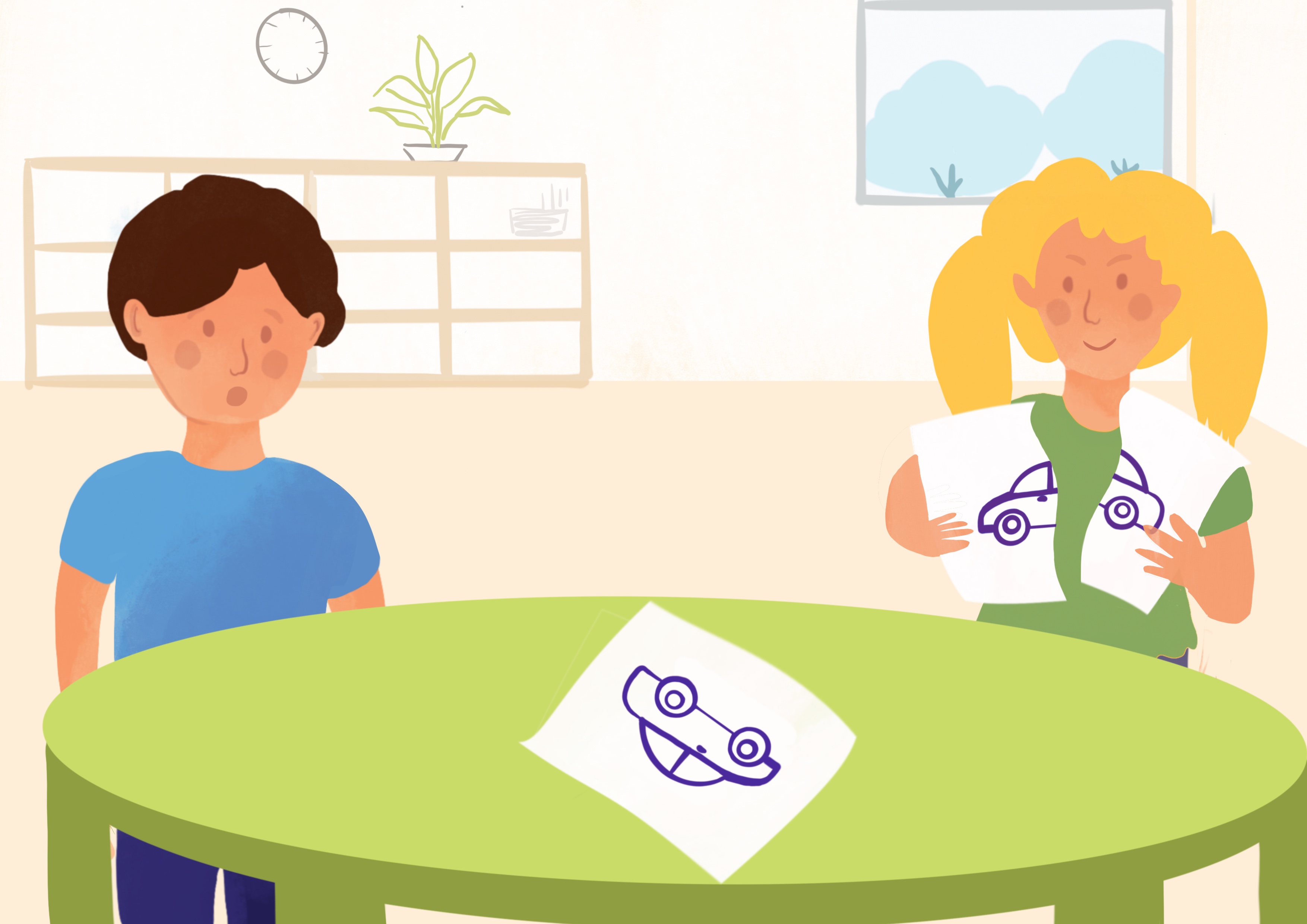 | 2.7. Lisa ripped the picture. She wanted to rip the picture. She did it on purpose. |
| 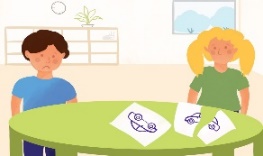 | 2.8. Tom looked at the ripped picture. “That was my picture!” said Tom. ***“I’m sorry, I was trying to see the picture better”*** said Lisa. Tom felt sad. | 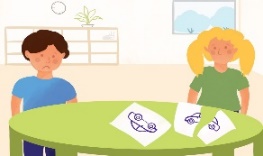 | 2.8. Tom looked at the ripped picture. “That was my picture!” said Tom. ***“I’m sorry, I thought your picture wasn’t good”*** said Lisa. Tom felt sad. |
| 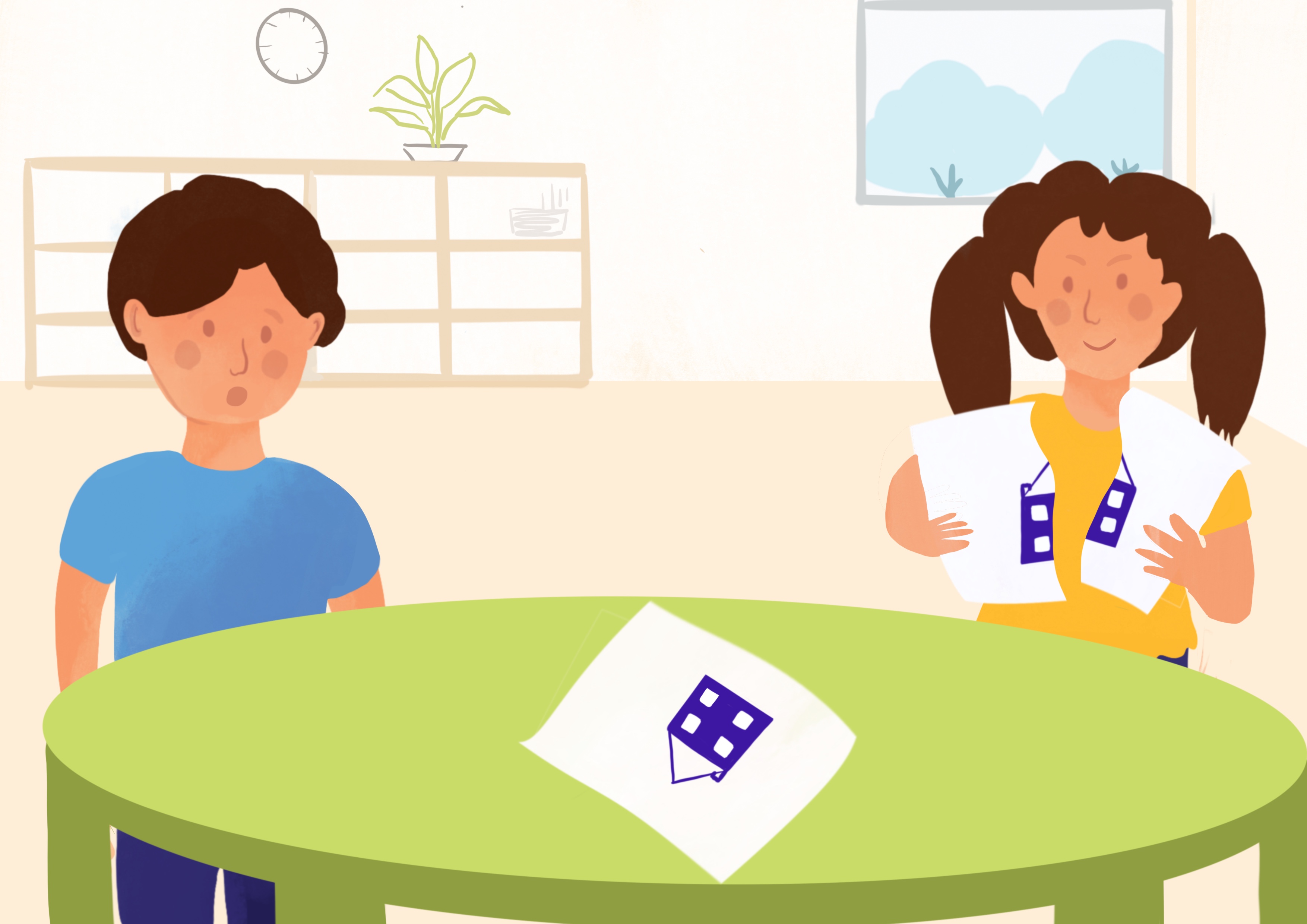 | 2.14. Poppy ripped the picture. She wanted to rip the picture. She did it on purpose. | 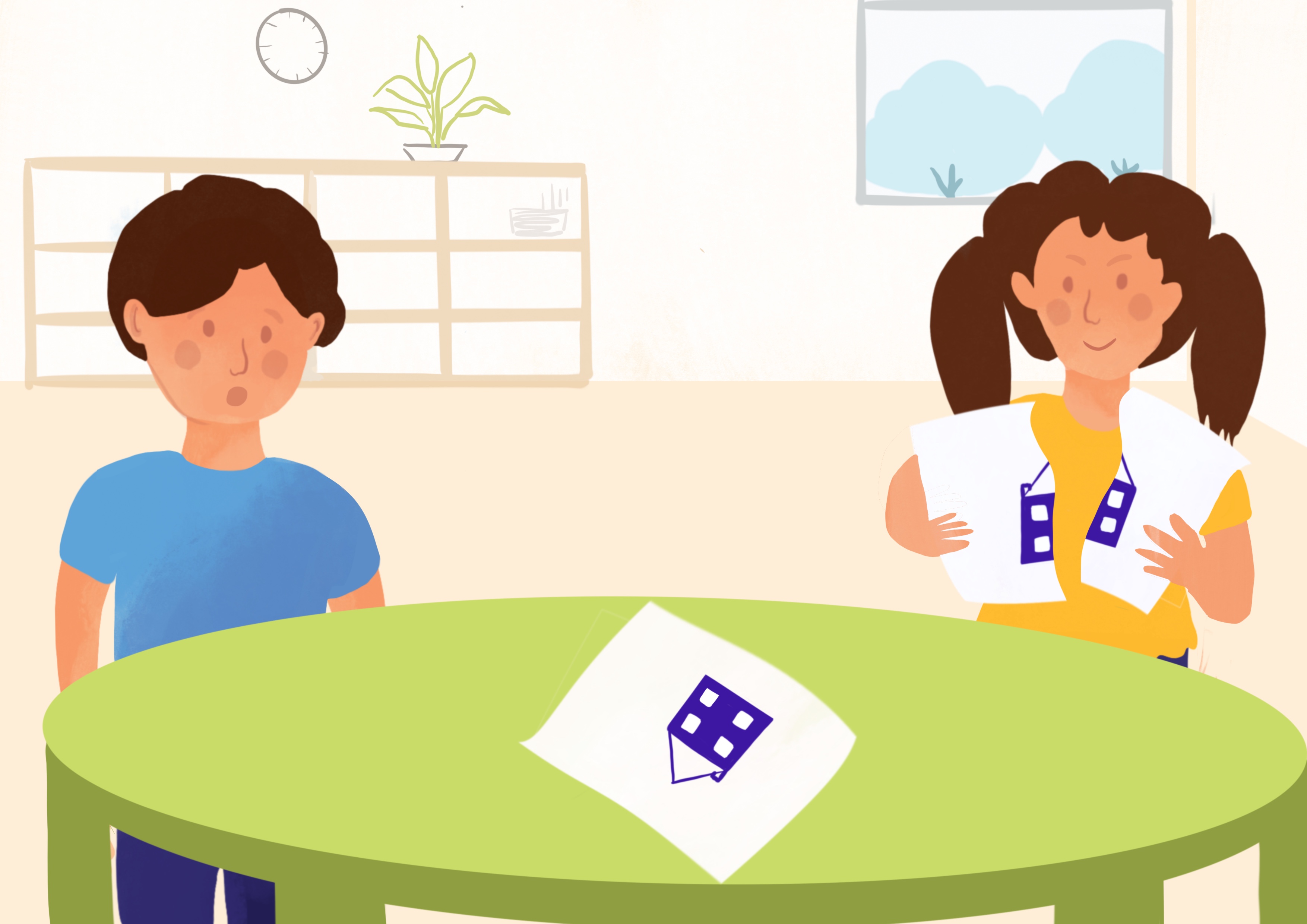 | 2.14. Poppy ripped the picture. She wanted to rip the picture. She did it on purpose. |
| 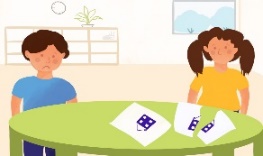 | 2.15. Tom looked at the ripped picture. “That was my picture!” said Tom. ***“I’m sorry,* *I thought this was my picture”*** said Poppy. Tom felt sad. | 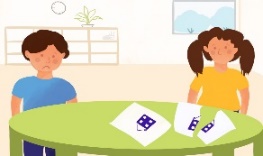 | 2.15. Tom looked at the ripped picture. “That was my picture!” said Tom. ***“I’m sorry,* *I thought this was my picture”*** said Poppy. Tom felt sad. |
