# Supplementary material for: Five‐year‐old children value reasons in apologies for belief‐based accidents
Source: Child Dev. 2023 Jan 24;94(3):e143–53. doi: 10.1111/cdev.13893 (PMC10952182; doi:10.1111/cdev.13893)
Supplement: Supplementary file 2 — Appendix S2. [file CDEV-94-e143-s001.docx]

**Supplementary Materials A**

***ANCOVA output summary for Study 1***:

ancova = aov (reason_preference ~ (age_cont * condition) + gender, data = apologies)

Anova (ancova, type = "III")

| ***Table 1.*** The fixed effects output for children’s overall preferences in Study 1 | | | | |
| --- | --- | --- | --- | --- |
|  | Sum Sq. | Df | F-value | *p*-value |
| (Intercept) | 81.512 | 1 | 123.0400 | <2e-16*** |
| Age | 0.248 | 1 | 0.3749 | 0.5419 |
| Condition | 0.393 | 1 | 0.5925 | 0.4434 |
| Gender | 0.479 | 1 | 0.7234 | 0.3973 |
| Age: Condition | 0.196 | 1 | 0.2953 | 0.5882 |
| Residuals | 60.286 | 91 |  |  |
| *Note.* *** *p* < .001. | | | | |

**Supplementary Materials B**

***GLM output summary for the “trust” question in Study 1***:

glm (trust ~ (age_cont * condition) + gender, data = apologies, family = binomial)

| ***Table 2.*** The fixed effects output for the “trust” question in Study 1 | | | | |
| --- | --- | --- | --- | --- |
|  | Estimate | Std.Err (SE) | z-value | *p*-value |
| (Intercept) | -0.0153 | 0.3796 | -0.040 | 0.9678 |
| Age | -0.3822 | 0.2942 | -1.299 | 0.1940 |
| Condition | 0.9985 | 0.4514 | 2.212 | 0.0270* |
| Gender | -0.2223 | 0.4475 | -0.497 | 0.6194 |
| Age: Condition | 1.0784 | 0.4836 | 2.230 | 0.0257* |
| *Note.* * *p* < .05. | | | | |

**Supplementary Materials C**

***GLM output summary for children’s justifications in Study 1***:

glm (justification ~ (age_cont * condition) + gender, data = apologies, family = binomial)

| ***Table 3.*** The fixed effects output for children’s justifications in Study 1 | | | | |
| --- | --- | --- | --- | --- |
|  | Estimate | Std.Err (SE) | z-value | *p*-value |
| (Intercept) | -2.5814 | 0.6360 | -4.059 | 4.93e-05*** |
| Age | -0.3311 | 0.4690 | -0.706 | 0.4802 |
| Condition | 0.7921 | 0.6676 | 1.187 | 0.2354 |
| Gender | 0.6199 | 0.6053 | 1.024 | 0.3058 |
| Age: Condition | 1.3278 | 0.6420 | 2.068 | 0.0386* |
| *Note.* * *p* < .05, *** *p* < .001. | | | | |

**Supplementary Materials D**

***Table 1.*** ANCOVA output summary for Study 1

Sum Sq. Df F-value *p*-value

(Intercept) 68.363 1 102.0959 <2e-16***

Age 0.244 1 0.3640 0.5478

Condition 0.397 1 0.5928 0.4433

Gender 0.426 1 0.6361 0.4272

Order 0.022 1 0.0330 0.8563

Age: Condition 0.194 1 0.2890 0.5922

Residuals 60.264 90

***Table 2.*** GLM output summary for the “trust” question in Study 1

Estimate Std.Err (SE) z-value *p*-value

(Intercept) 0.0833 0.4225 0.197 0.8437

Age -0.3767 0.2939 -1.282 0.2000

Condition 1.0080 0.4526 2.227 0.0259*

Gender -0.1777 0.4557 -0.390 0.6965

Order -0.2398 0.4506 -0.532 0.5946

Age: Condition 1.0857 0.4867 2.231 0.0257*

***Table 3.*** GLM output summary for children’s justifications in Study 1

Estimate Std.Err (SE) z-value *p*-value

(Intercept) -2.2604 0.6571 -3.440 0.0006***

Age -0.2722 0.4431 -0.614 0.5390

Condition 0.9033 0.6795 1.329 0.1837

Gender 0.8651 0.6404 1.351 0.1768

Order -1.1434 0.6452 -1.772 0.0764

Age: Condition 1.2552 0.6199 2.025 0.0429*

***Table 4.*** ANOVA output summary for Study 2

Sum Sq. Df F-value *p*-value

(Intercept) 5.863 1 7.8392 0.0076**

Condition 35.573 1 47.5605 1.622-08***

Gender 1.528 1 2.0429 0.1590

Order 1.126 1 1.5053 0.2264

Residuals 32.910 44

***Table 5.*** ANCOVA output summary for Study 2

Sum Sq. Df F-value *p*-value

(Intercept) 0.493 1 0.7588 0.3887

Age 0.763 1 1.1738 0.2848

Condition 0.870 1 1.3396 0.2536

Gender 3.010 1 4.6334 0.0372*

Order 1.363 1 2.0971 0.1550

Age: Condition 1.298 1 1.9980 0.1649

Residuals 27.287 42

***Table 6.*** GLM output summary for the “help” question in Study 2

Estimate Std.Err (SE) z-value *p*-value

(Intercept) 2.9135 1.1665 2.498 0.0125*

Condition -4.4872 1.2020 -3.733 0.0002***

Gender -0.4034 0.8854 -0.456 0.6487

Order 1.1651 0.9365 1.244 0.2135

***Table 7.*** GLM output summary for the “play” question in Study 2

Estimate Std.Err (SE) z-value *p*-value

(Intercept) 0.5698 0.6315 0.902 0.3669

Condition -2.1499 0.6945 -3.096 0.0020**

Gender 1.0103 0.6947 1.454 0.1459

Order 0.2616 0.6685 0.391 0.6956

***Table 8.*** GLM output summary for the “trust” question in Study 2

Estimate Std.Err (SE) z-value *p*-value

(Intercept) 1.5285 0.8307 1.840 0.0658

Condition -3.3323 0.9593 -3.474 0.0005***

Gender 1.4712 0.8370 1.758 0.0788

Order 0.9334 0.8009 1.165 0.2438

***Table 9.*** GLM output summary for children’s justifications in Study 2

Estimate Std.Err (SE) z-value *p*-value

(Intercept) -1.6246 0.7014 -2.316 0.0206*

Condition 1.8601 0.6616 2.811 0.0049**

Gender 0.1381 0.6509 0.212 0.8320

Order 0.8375 0.6603 1.268 0.2046

Note. * *p* < .05, ** *p* < .01, *** *p* < .001

**Supplementary Materials E**

***ANOVA output summary for Study 2***:

anovaS2 = aov (belief_preference ~ condition + gender, data = apologies2)

Anova (anovaS2, type = "III")

| ***Table 4.*** The fixed effects output for children’s overall preferences in Study 2 | | | | |
| --- | --- | --- | --- | --- |
|  | Sum Sq. | Df | F-value | *p*-value |
| (Intercept) | 4.798 | 1 | 6.3436 | 0.0154* |
| Condition | 35.551 | 1 | 47.0034 | 1.667e-08*** |
| Gender | 1.423 | 1 | 1.8813 | 0.1770 |
| Residuals | 34.035 | 45 |  |  |
| *Note.* * *p* < .05, *** *p* < .001. | | | | |

**Supplementary Materials F**

***ANCOVA output summary for Study 2***:

ancovaS2 = aov (belief_preference ~ (age_cont * condition) + gender, data = apologies2)

Anova (ancovaS2, type="III")

| ***Table 5.*** The fixed effects output for children’s overall preferences in Study 2 | | | | |
| --- | --- | --- | --- | --- |
|  | Sum Sq. | Df | F-value | *p*-value |
| (Intercept) | 1.099 | 1 | 1.6503 | 0.2058 |
| Age | 1.388 | 1 | 2.0830 | 0.1562 |
| Condition | 0.410 | 1 | 0.6152 | 0.4372 |
| Gender | 2.792 | 1 | 4.1909 | 0.0468* |
| Age: Condition | 0.729 | 1 | 1.0940 | 0.3014 |
| Residuals | 28.649 | 43 |  |  |
| *Note.* * *p* < .05. | | | | |

**Supplementary Materials G**

***GLM output summary for the “help”, “play” and “trust” questions in Study 2***:

**Help:** glm (help ~ condition + gender, data = apologies2, family = binomial)

| ***Table 6.*** The fixed effects output for the “help” question in Study 2 | | | | |
| --- | --- | --- | --- | --- |
|  | Estimate | Std.Err (SE) | z-value | *p*-value |
| (Intercept) | 3.3366 | 1.1268 | 2.961 | 0.003064** |
| Condition | -4.2456 | 1.1296 | -3.759 | 0.000171*** |
| Gender | -0.3991 | 0.8631 | -0.462 | 0.643790 |
| *Note.* ** *p* < .01, *** *p* < .001. | | | | |

**Play:** glm (play ~ condition + gender, data = apologies2, family = binomial)

| ***Table 7.*** The fixed effects output for the “play” question in Study 2 | | | | |
| --- | --- | --- | --- | --- |
|  | Estimate | Std.Err (SE) | z-value | *p*-value |
| (Intercept) | 0.7039 | 0.5338 | 1.319 | 0.18723 |
| Condition | -2.1420 | 0.6924 | -3.094 | 0.00198** |
| Gender | 0.9970 | 0.6925 | 1.440 | 0.14999 |
| *Note.* ** *p* < .01. | | | | |

**Trust:** glm (trust ~ condition + gender, data = apologies2, family = binomial)

| ***Table 8.*** The fixed effects output for the “trust” question in Study 2 | | | | |
| --- | --- | --- | --- | --- |
|  | Estimate | Std.Err (SE) | z-value | *p*-value |
| (Intercept) | 1.9459 | 0.7667 | 2.538 | 0.011153* |
| Condition | -3.2189 | 0.9274 | -3.471 | 0.000519*** |
| Gender | 1.3981 | 0.8152 | 1.715 | 0.086325 |
| *Note.* * *p* < .05, *** *p* < .001. | | | | |

**Supplementary Materials H**

***GLM output summary for children’s justifications in Study 2***:

glm (justification ~ condition + gender, data = apologies2, family = binomial)

| ***Table 9.*** The fixed effects output for children’s justifications in Study 2 | | | | |
| --- | --- | --- | --- | --- |
|  | Estimate | Std.Err (SE) | z-value | *p*-value |
| (Intercept) | -1.1460 | 0.5593 | -2.049 | 0.04045* |
| Condition | 1.7886 | 0.6404 | 2.793 | 0.00522** |
| Gender | 0.1020 | 0.6385 | 0.160 | 0.87314 |
| *Note.* * *p* < .05, ** *p* < .01. | | | | |
